# Highly substituted benzannulated cyclooctanol derivatives by samarium diiodide-induced cyclizations

**DOI:** 10.3762/bjoc.6.141

**Published:** 2010-12-28

**Authors:** Jakub Saadi, Irene Brüdgam, Hans-Ulrich Reissig

**Affiliations:** 1 Freie Universität Berlin, Institut für Chemie und Biochemie, Takustrasse 3, D-14195 Berlin, Germany

**Keywords:** cyclooctanes, ketones, ketyls, medium-sized rings, samarium diiodide, single electron transfer, styrene derivatives, radicals

## Abstract

A series of γ-oxo esters suitably substituted with various styrene subunits was subjected to samarium diiodide-induced 8-*endo*-*trig* cyclizations. Efficacy, regioselectivity and stereoselectivity of these reactions via samarium ketyls strongly depend on the substitution pattern of the attacked alkene moiety. The stereoselectivity of the protonation of the intermediate samariumorganyl is also influenced by the structural features of the substrates. This systematic study reveals that steric and electronic factors exhibited by the alkene and ketone subunits are of high importance for the outcome of these cyclization reactions leading to highly substituted benzannulated cyclooctanol derivatives. In exceptional cases, 7*-exo-trig* cyclizations to cycloheptanol derivatives have been observed. In examples with high steric hindrance the ketyl–aryl coupling can be a competing process.

## Introduction

**Scheme 1:**
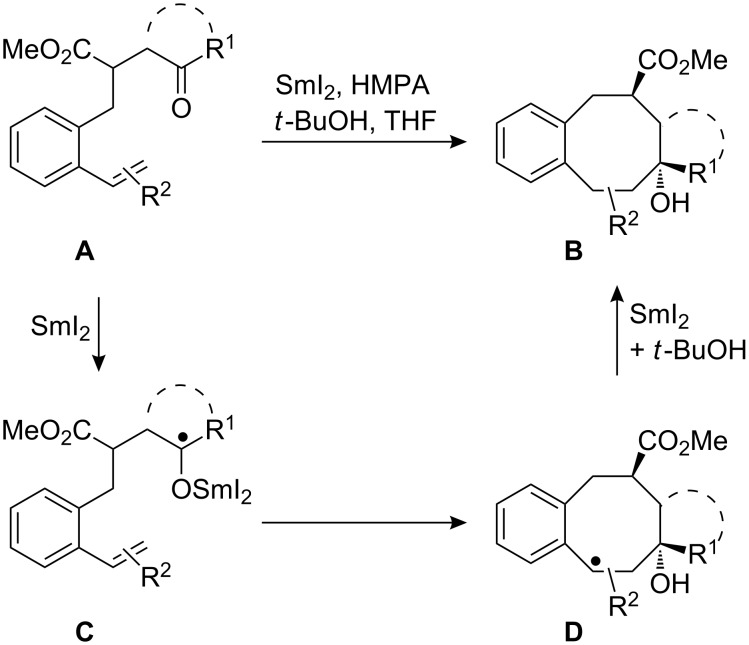
SmI_2_-induced cyclizations of styryl-substituted γ-ketoesters **A** to benzannulated cyclooctanol derivatives **B** via samarium ketyl **C** and radical **D** (HMPA ligands at the samarium are omitted for simplicity in all Schemes, but certainly can play an important role for the outcome).

Functionalized cyclooctane substructures are frequently found in natural products and pharmacologically significant compounds. Because of their potential biological activity and intriguing geometrical features, the construction of cyclooctanoid frameworks has challenged synthetic organic chemists for a long time [1–2]. This task is quite challenging due to unfavourable enthalpic and entropic factors during the formation of medium-sized rings [3]. Nevertheless, in recent years a range of interesting solutions for the efficient formation of eight-membered rings has been developed [1–2,4–18]. Successful approaches include ring-closing metathesis [4], rearrangements [5], and cycloadditions [6], transition metal-catalyzed cyclizations [7–8], nucleophilic and electrophilic substitution reactions [9] as well as ring expansion reactions [10]. Among these approaches to carbocyclic compounds, samarium diiodide-mediated reactions play an important role and have been described in a number of excellent review articles [19–21] and original publications [22–35]. In our previous reports we have described the efficient synthesis of cyclooctanol and cyclooctenol derivatives by samarium diiodide-induced 8-*endo*-*trig* and 8-*endo*-*dig* cyclizations of γ-styryl- [20,31,33] and γ-phenylalkynyl-substituted [20,32] ketoesters. Recently, we have also reported our preliminary results on cyclizations of analogous starting materials bearing alkyl and aryl substituents at the styryl double bond, which furnished highly substituted cyclooctanol derivatives (Scheme 1) and in some cases compounds with cycloheptanol substructure [34]. The transformation of **A** to **B** proceeds via the samarium ketyl **C** followed by the 8*-endo-trig* cyclization to **D**. Subsequent reduction of the radical **D** by the second equivalent samarium diiodide and protonation furnishes the cyclooctanol derivative **B**. The details of this mechanism have been discussed earlier [31]. This method was also successfully applied to the synthesis of cyclic compounds larger than cyclooctane derivatives [35]. Here, we would like to present our detailed results showing the scope and limitations of this method to the synthesis of highly substituted cyclooctanol derivatives and the influence of the substitution pattern of the precursors on the regioselectivity and stereochemical outcome. First, the impact on the reaction by alkyl and aryl substituents at the α-styryl carbon of the substrates will be described, then we discuss the influence of analogous β-styryl substituents.

## Results and Discussion

**Scheme 2:**
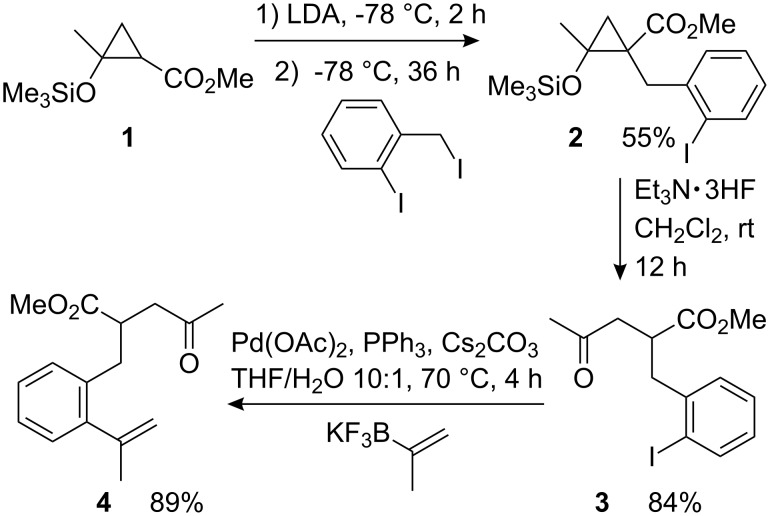
Three-step synthesis of precursor **4** starting from siloxycyclopropane derivative **1**.

Starting materials were prepared from readily available siloxycyclopropanes in analogy to our previously described modular approach [33]. As a typical example, the synthesis of precursor **4** is depicted in Scheme 2. Cyclopropane **1** [36–37] was deprotonated with LDA and subsequently alkylated with 2-iodobenzyl iodide, furnishing **2** in moderate yield. Intermediate **2** was then treated with triethylamine trihydrofluoride, furnishing 2-iodobenzyl-substituted γ-ketoester **3**. The following palladium-catalyzed cross-coupling [38–39] with potassium 2-propenyl trifluoroborate afforded the cyclization precursor **4** in very good yield.

**Scheme 3:**
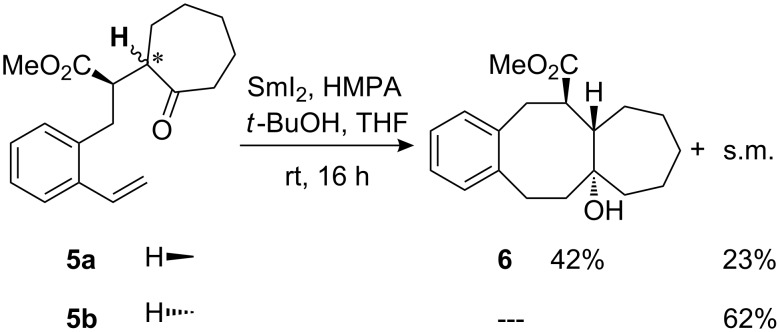
Attempted cyclizations of diastereomeric cycloheptanone derivatives **5a** and **5b**.

The cyclization reactions were generally performed with 2.2 equiv of samarium diiodide in THF and in the presence of 18 equiv of HMPA and 2 equiv of *tert*-butanol. HMPA is crucial for the success of most of the described cyclizations because of its unique influence on the reactivity of samarium diiodide [40–44]. For control experiments and an add-on to our previous observations [33], we started our experiments with the cyclization of ketoesters **5a**/**b** containing a cycloheptanone subunit. Here only the *unlike*-configured [45] **5a** furnished the expected benzannulated tricyclic compound **6** in moderate yield (Scheme 3). The *like*-configured precursor **5b** is unable to arrange the reacting moieties (carbonyl group and alkene) in appropriate proximity while retaining an energetically favourable conformation of the molecule. The impact of the relative configuration of cyclic ketones as starting materials on the 8-*endo*-*trig* process is in full agreement with our previous observations for the corresponding cyclohexanone derivatives [33].

**Scheme 4:**
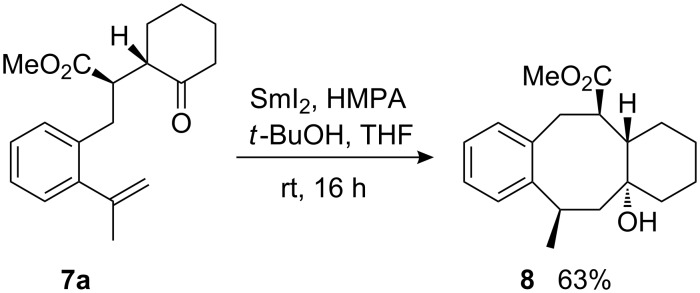
Samarium diiodide-induced cyclization of γ-ketoester **7a** to tricyclic compound **8**.

Because of the observed sensitivity of this reaction to steric factors, it was necessary to study the tolerance and limitations for the substitution pattern of the starting materials in more detail. At first, the influence of a methyl substituent at the α-styryl carbon was investigated. Cyclization of the *unlike*-configured cyclohexanone derivative **7a** afforded the expected tricyclic cyclooctanol derivative **8** in good yield and excellent *trans*-stereoselectivity (Scheme 4). The terms *cis* and *trans* refer in this report to the position of the methoxycarbonyl group and the hydroxyl group. In general, *cis*-configured products directly undergo a subsequent cyclization to the corresponding γ-lactones. It is noteworthy that the additionally introduced stereogenic centre bearing the methyl group is also generated stereoselectively.

In part, the configuration of **8** was assigned based on the relative configuration of the starting material. The lack of lactone formation indicates that the bridgehead hydroxyl group is *trans* located with respect to the methoxycarbonyl group. The configuration at the methyl-substituted stereocenter was assigned based on NOESY-correlations and careful comparison of spectroscopic data with those of related compounds. The analogous cyclopentanone-derived starting material was also tested in the cyclization reaction; however, it afforded a complex mixture of products.

**Scheme 5:**
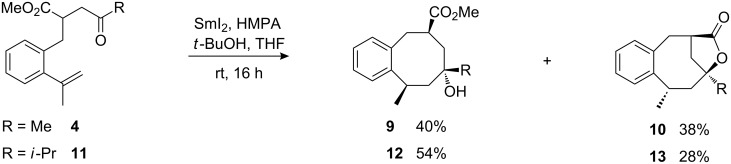
Samarium diiodide-induced cyclizations of methyl ketone **4** and *iso*-propyl ketone **11**.

Cyclization of acyclic starting material **4**, bearing a methyl substituent at the α-styryl carbon, furnished a mixture of *trans*- and *cis*-cyclization products **9** and **10** in very good combined yield, but practically no stereochemical preference. The precursor **11**, bearing the more bulky *iso*-propyl ketone subunit, provided cyclooctanol derivatives **12** and **13** with similarly good combined yield and a clear preference for *trans*-product **12** (Scheme 5).

**Figure 1:**
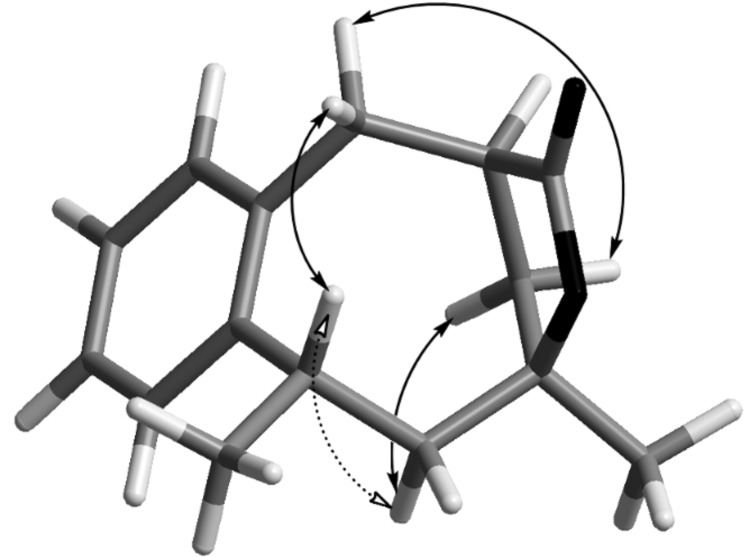
NOESY-correlation for compound **10**.

The relative configurations of lactone-bridged compounds **10** and **13** were deduced from NOESY-correlations and the assumption was confirmed that the five-membered lactone bridge, which is quite constrained and rigid, can be formed only when carboxyl and hydroxyl groups are at the same face of the eight-membered ring (Figure 1). This analysis was further supported by high similarity of NMR-spectroscopic data (^1^H and ^13^C NMR shifts and relative signal patterns) with those of compound **17a** and at the same time rather big differences to those of the epimeric compound **17b**.

**Figure 2:**
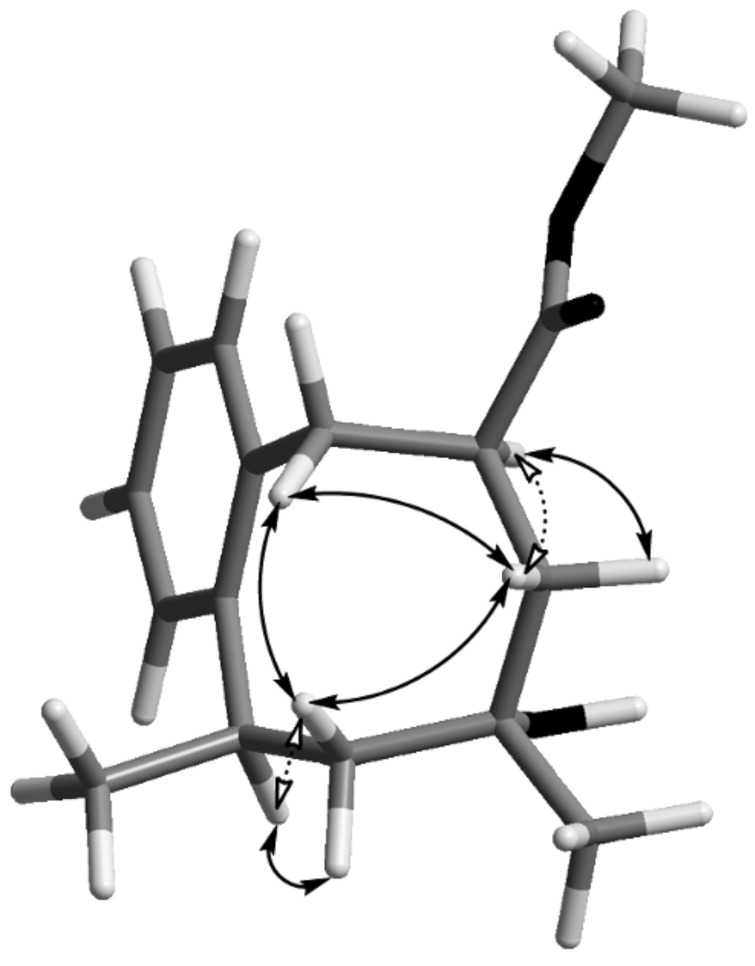
NOESY-correlation for compound **9**.

The NOESY-experiments also indicated the proton correlations across the eight-membered ring in compounds **9** and **12**. The assignment was further supported by the correlations between the protons at carbons with one substituent at the ring and their neighbouring protons. The *cis*-vicinal protons indicated strong correlations, while the *trans*-vicinal protons, where the dihedral angle was close to 180°, show none or almost no correlations between each other (Figure 2).

**Scheme 6:**
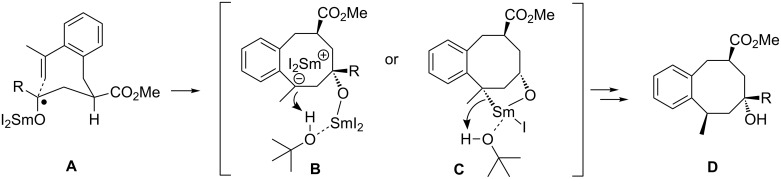
Assumed transition structures and intermediates **A**, **B**, or **C** for the cyclizations of (2-propenyl)phenyl-substituted ketones **4** and **11** leading to products of type **D** (HMPA ligands at the samarium are omitted for simplicity).

In cyclizations of the (2-propenyl)phenyl-substituted ketoesters, *trans*-products are preferred; however the *cis*/*trans* stereoselectivity was generally lower than that observed for the cyclizations of their styrene analogues [33]. It seems that the previously observed correlation between the bulkiness of a ketone subunit and the increase in stereoselectivity is also effective here. This result is in accordance to the proposed eight-membered pseudo-chair-like transition structure [33], where the methoxycarbonyl group occupies a preferred pseudo-equatorial position and the ketyl substituent is competing with the bulky samariumoxy group for the other pseudo-equatorial position. Therefore, ketones with bulkier substituents preferentially react through a transition structure **A** (Scheme 6) leading to the *trans*-cyclization product. Remarkably, the orientation of the methyl group at the newly formed third stereogenic centre, which arises during the protonation of the samariumorganyl intermediate, was always *trans* to the hydroxyl group, arising from the ketone moiety. This interesting observation indicates that the protonation is highly stereoselective and the protons are coming from the same face of the ring where the samariumoxy group is situated after the completed cyclization. Since samarium(III) is very oxophilic, it is possible that the samariumoxy group coordinates *tert*-butanol, which is then a more acidic proton source (intermediates **B** and **C**, Scheme 6). This template effect would then be responsible for the stereoselective protonation governed by the samariumoxy moiety. An intermediate similar to **C** (covalent C–Sm bond or contact ion pair) may be a plausible alternative, which is then protonated under retention by the proton source [46].

**Scheme 7:**
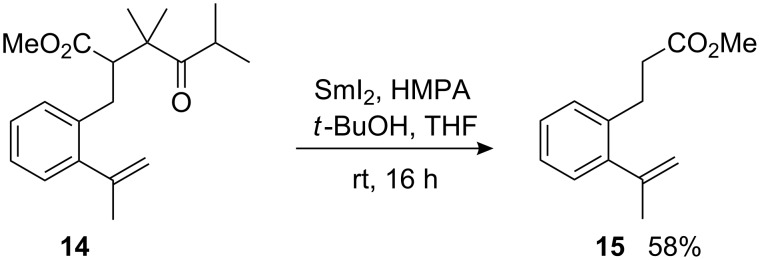
Reductive fragmentation of highly hindered ketoester **14**.

The introduction of two additional methyl substituents between the ketone and ester moieties in the bulky diisopropyl ketone derived substrate **14**, suppressed the 8-*endo*-*trig* cyclization completely and only the fragmentation product **15** was isolated from the reaction mixture (Scheme 7). It is not clear whether the mechanism of SmI_2_-induced fragmentation of 1,4-dicarbonyl compounds is of anionic or radical nature, but it has been observed in several cases [33,47–50]. It is possible that, due to the high steric hindrance of the initially formed samarium ketyl, the coupling to the alkene is very slow. The ketyl or the anion formed by its further reduction with SmI_2_ fragments to the samarium enolate of diisopropyl ketone and the radical or anion stabilized by the methoxycarbonyl group. Very surprisingly in this reaction, small amounts of the *n*-propyl ester of **15** were also found. At this moment, the origin of the *n*-propyl group is not clear.

**Scheme 8:**
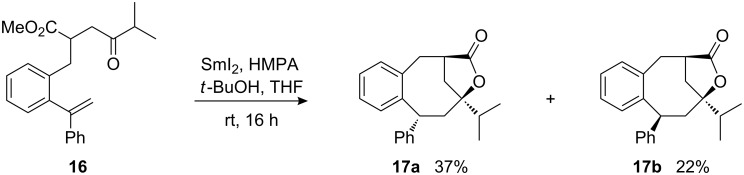
Samarium diiodide-induced cyclization of phenyl-substituted substrate **16** leading to lactones **17a** and **17b**.

The modulation analysis of the electronic properties of the reacting alkene was evaluated by the introduction of a phenyl substituent to the α-styryl position. The samarium diiodide-induced cyclization of ketoester **16** afforded two epimeric lactone bridged *cis*-products, **17a** and **17b**, in ca. 1.7:1 ratio and in good combined yield (Scheme 8).

**Figure 3:**
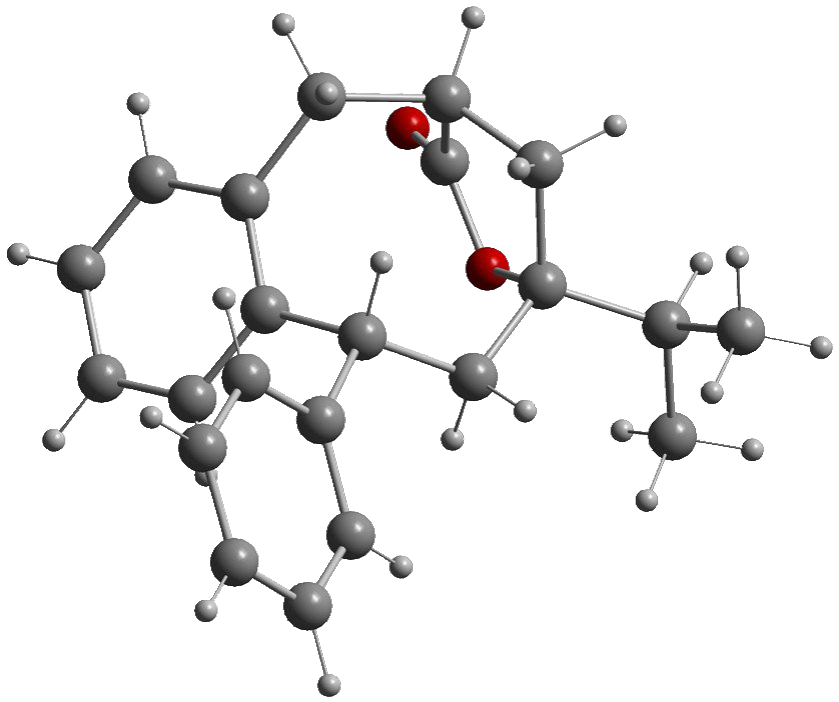
Molecular structure (Diamond [52]) of compound **17b**.

The relative configuration of **17b** was unambiguously assigned by X-ray crystal structure (Figure 3) [51]. Assuming that the lactone bridge is formed only when carboxyl and hydroxyl groups are at the same face of the eight-membered ring, the configuration of **17a** with the inverted phenyl-substituted stereocenter was assigned as the only alternative to **17b**.

The exclusive *cis-*selectivity of cyclization, observed in this case, may be attributed to a high level of the steric repulsions between the bulky phenyl group at the alkene moiety and the *iso*-propyl group at the samarium ketyl in the usually preferred *trans*-transition structure (compare **A**, Scheme 6). It is unclear, why the protonation of the samariumorganyl species, which is additionally stabilized by the phenyl substituent, occurred with lower stereochemical preference in this case. However, if an intermediate such as **C** (Scheme 6) is also involved in this transformation a less covalent C-Sm bond may lead to a decreased stereoselectivity of the protonation step.

**Scheme 9:**
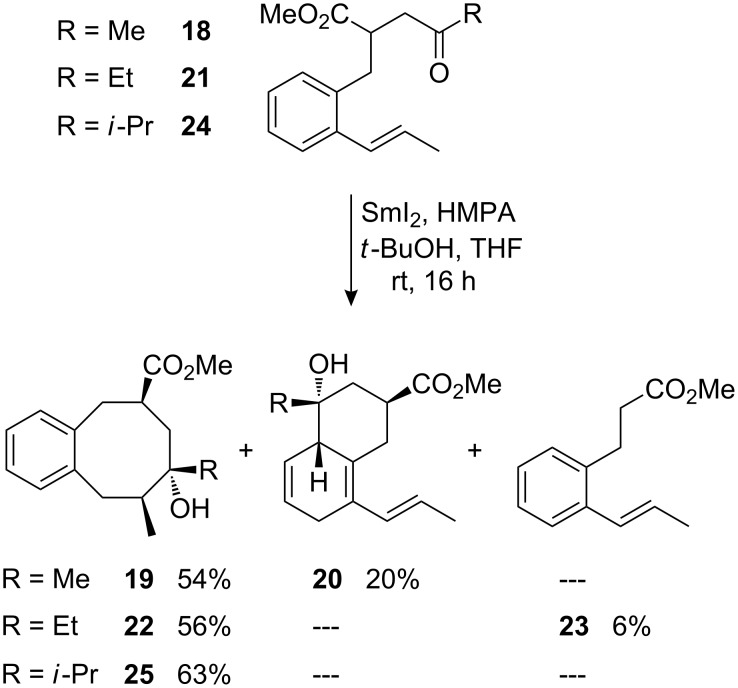
Samarium diiodide-induced cyclizations of (*E*)-(1-propenyl)phenyl-substituted γ-ketoesters **18**, **21**, and **24**.

We have subsequently investigated the influence of alkyl substituents at the β-styryl carbon. In this series, (*E*)-1-propenyl-substituted acyclic ketones **18**, **21**, and **24** gave the expected *trans*-cyclooctanol derivatives **19**, **22**, and **25** in moderate to good yields and with excellent stereoselectivities (Scheme 9). In case of cyclization of methyl-substituted ketone **18**, the side-product **20** was obtained as a result of a ketyl–aryl coupling [20,53–58] in addition to the desired cyclooctanol derivative **19**. Also minor amounts of fragmentation product **23** were detected in the cyclization of ethyl ketone **21** (Scheme 9). These two observations indicate that the transition structures of the cyclizations leading to eight-membered rings are more constrained and that the reactions are slower than those where a terminally unsubstituted alkene is attacked.

The assignment of the configuration of product **20** is based on that of numerous earlier described examples of SmI_2_-induced ketyl–aryl coupling products [57]. The configurations of products **19**, **22**, and **25** were assigned on the basis of NOESY-correlations.

**Figure 4:**
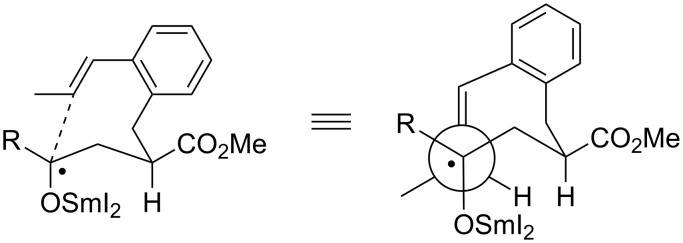
Proposed transition structure for the cyclization of (*E*)-1-propenyl-substituted substrates (HMPA ligands and proton donors ROH at the samarium are omitted for simplicity).

The transition structure for the SmI_2_-mediated 8-*endo*-*trig* cyclizations of these (*E*)-1-propenyl-substituted substrates is proposed in accordance to the previously postulated eight-membered pseudo-chair-like transition structures (Figure 4). The methoxycarbonyl group and substituent R at the ketyl moiety are both occupying preferred pseudo-equatorial positions. In our drawing, the olefin approaches the samarium ketyl from the backside perpendicularly to the bulky samariumoxy group. The methyl substituent at the alkene moiety prefers a staggered position in between the bulky samariumoxy group and substituent R.

**Scheme 10:**
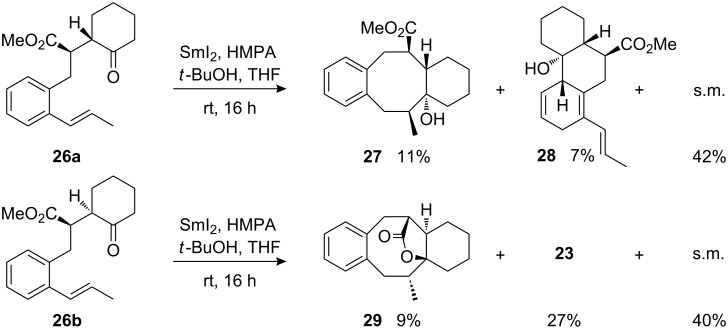
Attempted samarium diiodide-induced cyclizations with (*E*)-1-propenyl-substituted precursors **26a** and **26b**.

Another indication of the considerable steric repulsion introduced by the methyl substituent at the β-styryl position is provided by the cyclizations of sterically more constrained cyclohexanone derivatives **26a** and **26b** (Scheme 10). In these two cases both the usually preferred *unlike*-configured precursor **26a** and the disfavoured *like*-configured **26b** gave only low quantities of cyclooctanol products, **27** and **29**, respectively, along with the recovered starting material and either the ketyl-aryl coupling product **28** or the fragmentation product **23** (Scheme 10).

The stereochemical assignments for products **27** and **29** are based on the relative configuration of their precursors. The existence or lack of lactone formation allows for assigning the configuration of the bridgehead carbon bearing the hydroxyl group. The assignment of the centre with the methyl group is based on a strong NOESY-correlation between the methyl protons and the bridgehead proton. It is noteworthy that the stereoselectivity of the cyclization for cyclic ketones is clearly controlled by the relative configuration of the precursors and that the hydroxyl group is generated *trans* to the vicinal substituents. The reactivity of the sterically more demanding β-styryl-substituted substrates was strongly retarded due to rigidity and bulkiness of the integrated cyclohexane ring, which in the previous cyclization examples afforded cyclooctanol products with good yield and excellent stereoselectivity.

The cyclopentanone-derived analogues of **26a**/**b** were also examined in this samarium diiodide-promoted transformation, but among the isolated products only the starting materials could be unequivocally identified. Not surprisingly, the (*E*)-1-propenyl analogue of the bulky diisopropyl ketone-derived substrate **14** only afforded the corresponding fragmentation product. In this case again small amounts of the *n*-propyl ester were detected (compare Scheme 7).

**Scheme 11:**
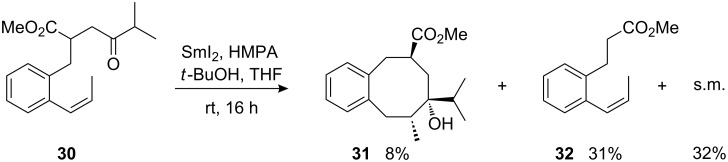
Attempted samarium diiodide-induced cyclization of (*Z*)-1-propenyl-substituted precursor **30**.

The influence of the configuration of the reacting alkene was also studied. The cyclization of (*Z*)-1-propenyl-substituted **30**, which is isomeric to already examined (*E*)-1-propenyl-ketoester **24**, afforded the corresponding cyclooctanol **31** with considerably lower efficacy (Scheme 11, compare Scheme 9). The reaction mixture afforded considerable amounts of fragmentation product **32** along with unchanged starting material.

The configuration of product **31** is based on the comparison with the isomeric product **25**. Analysis of a possible transition structure analogous to that proposed for the cyclization of (*E*)-1-propenyl-substituted γ-ketoesters (compare Figure 4) reveals that the *Z*-configured alkene has to arrange the methyl substituent in a highly unfavourable *endo*-cyclic position of the newly formed ring. This apparently causes high steric repulsion and thus strongly suppressed reactivity.

**Scheme 12:**
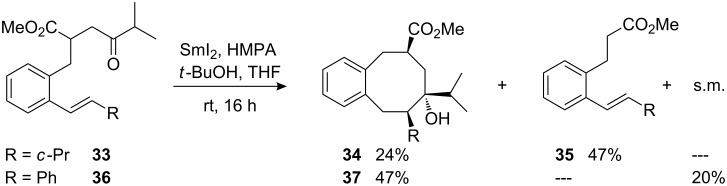
Samarium diiodide-induced cyclizations of γ-ketoesters **33** and **36**.

In order to further define the scope and limitations of the samarium diiodide-induced reaction we then studied the cyclization of β-styryl-substituted γ-ketoester **33**. This precursor has the preferred (*E*)-configured alkene substructure, but is equipped with branched substituents at the ketone and alkene moieties (Scheme 12). The starting material was completely consumed in this reaction, affording a moderate yield of the eight-membered carbocycle **34** together with a fairly high amount of fragmentation product **35**. The obtained product ratio suggests that in this example, the cyclization is roughly two times slower than the fragmentation. When a phenyl group was introduced to the β-styrene position the cyclization of γ-ketoester **36** mainly afforded the desired cyclooctanol derivative **37**, along with several undefined side products in low quantities and 20% of unconsumed starting material (Scheme 12). It has to be emphasized here, that the 1,2-diarylalkene unit can potentially react with the samarium ketyl at both carbon atoms affording two different stabilized benzylic radicals. Therefore it is an interesting observation that the 8-*endo*-*trig* cyclization mode is preferred over the possible 7-*exo*-*trig* mode for the reaction of compound **36**.

The configurations of products **34** and **37** are assigned with the aid of NOESY-correlations and by comparison with the NMR-spectroscopic data of analogous compounds obtained after cyclizations of (*E*)-1-propenyl-substituted γ-ketoesters.

**Scheme 13:**
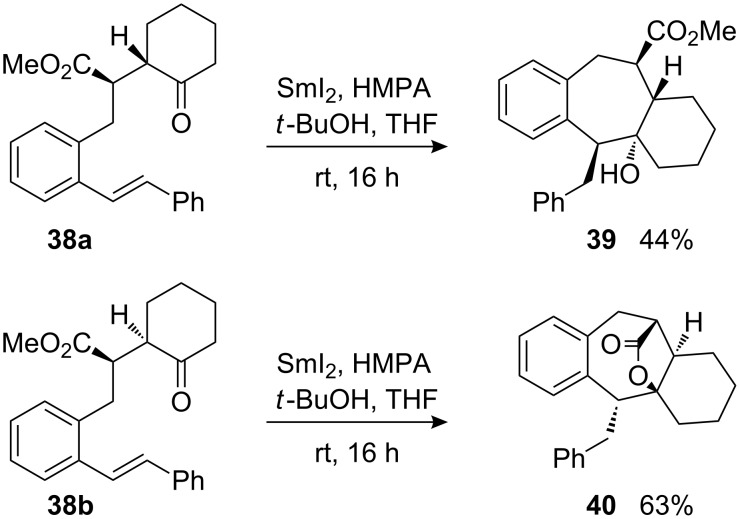
Samarium diiodide-induced cyclizations of diastereomeric stilbenyl-substituted γ-ketoesters **38a** and **38b**.

In contrary to the reaction of acyclic ketone **36**, stilbenyl-substituted cyclohexanone derivatives **38a** and **38b** both preferred to undergo a 7-*exo*-*trig* cyclization, yielding tricyclic cycloheptanol derivative **39** and tetracyclic lactone **40**, respectively (Scheme 13). Similarly as in cyclizations of the cyclohexanone-derived ketoesters with terminally substituted alkenes **26a**/**b**, the 8-*endo*-*trig* cyclization pathway may be quite hampered in the cases of **38a** and **38b**. However, due to the radical stabilizing properties of the terminal phenyl substituent, the 7-*exo*-*trig* pathway is now possible. It is probable that the restricted conformational flexibility of the cyclic ketones leads to higher steric and torsional strain in an alternative eight-membered transition structure. This assumption is supported by the observation that conformationally more flexible acyclic γ-ketoesters, such as **36**, prefer the 8-*endo*-*trig* cyclization mode. Furthermore, the *like*-configured starting material **38b**, which is disfavoured in 8-*endo*-*trig* cyclizations, afforded the cycloheptanol derivative with even better efficacy than the *unlike*-configured **38a**.

**Figure 5:**
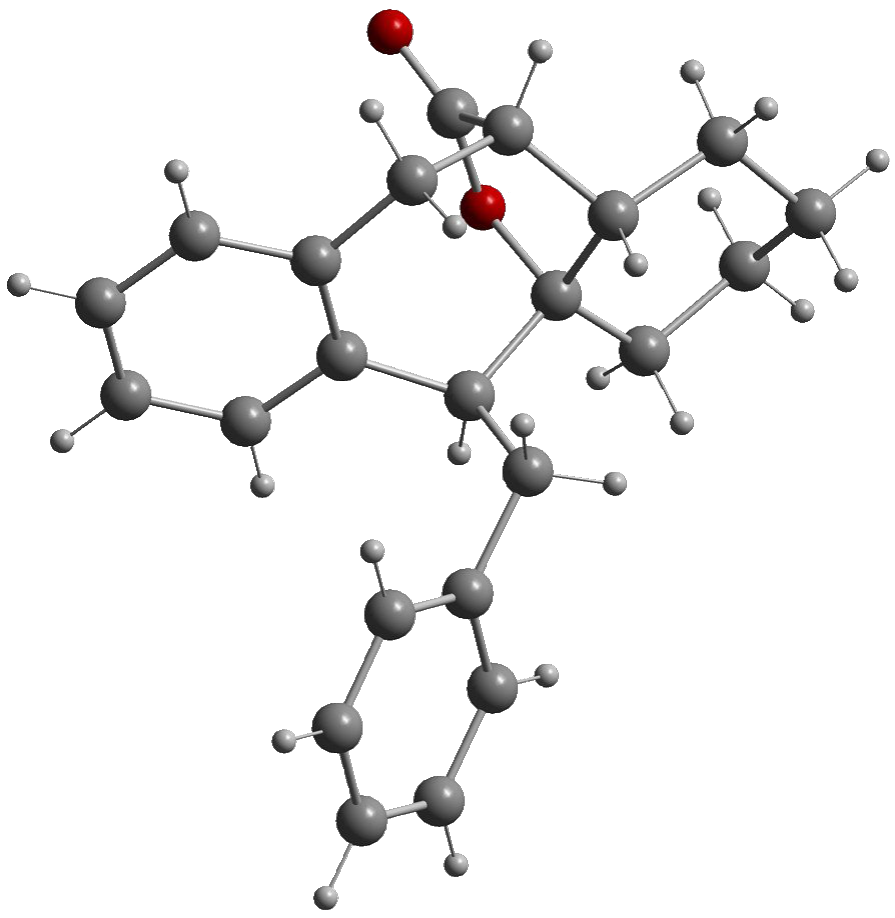
Molecular structure (Diamond [52]) of compound **40**.

The constitution and the relative configuration of compound **40**, featuring a tetracyclic lactone-bridged core, were unambiguously determined by an X-ray crystal structure (Figure 5) [59]. The constitution of compound **39** was assigned as a cycloheptanol derivative based on the similarities of its ^13^C NMR data with those of **40** rather than those of the analogous eight-membered carbocycles. The configuration of **39** was assigned by using the following arguments: the relative configuration of the *unlike*-configured precursor should be transferred to the product and the bridgehead hydroxyl group should be in *trans*-relationship to the methoxycarbonyl group since no lactone formation was observed. The configuration of the stereogenic centre with the phenyl group of **39** is based on NOESY-correlations between the exocyclic benzylic protons and the bridgehead proton.

**Scheme 14:**
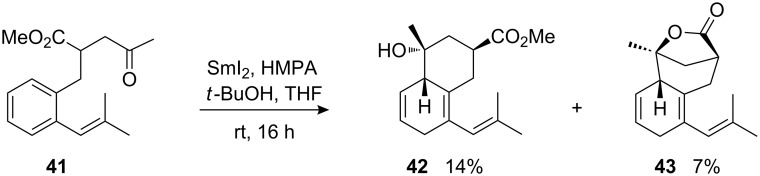
Attempted cyclization of β-dialkyl-substituted styrene derivative **41**.

An attempt to cyclize γ-ketoester **41** featuring a β-dialkyl-substituted styryl moiety furnished only minor amounts of the ketyl–aryl coupling products **42** and **43** (Scheme 14). Similar to some other reactions leading mainly to side products, trace amounts of *n*-propyl ester of **42** of unknown origin were detected. Although the precursor was completely consumed, the missing material could not be identified. The relative configuration of the hexahydronaphthalene derivatives was assigned by the comparison of spectroscopic data with that of the previously described analogous compounds [57].

## Conclusion

**Scheme 15:**
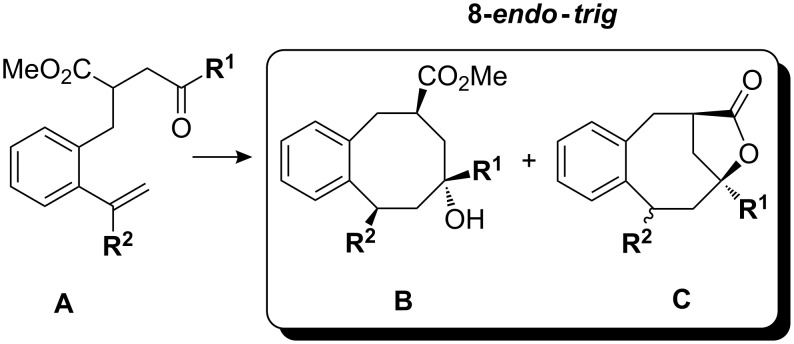
Typical products of samarium diiodide-induced 8-*endo*-*trig* cyclizations of α-styryl-substituted γ-ketoesters.

Samarium diiodide-mediated 8-*endo*-*trig* cyclizations of styryl-substituted γ-ketoesters bearing alkyl or aryl substituents at the α- or β-styryl carbon were systematically studied. The stereoselectivity of these transformations was strongly influenced by the steric bulk at the ketone and alkene moieties. Acyclic γ-ketoesters such as **A** cyclized very efficiently, affording mixtures of *cis*- and *trans*-products **B** and **C** with improving *cis*/*trans* stereoselectivity by increase of the size of the ketone substituents (Scheme 15).

**Scheme 16:**
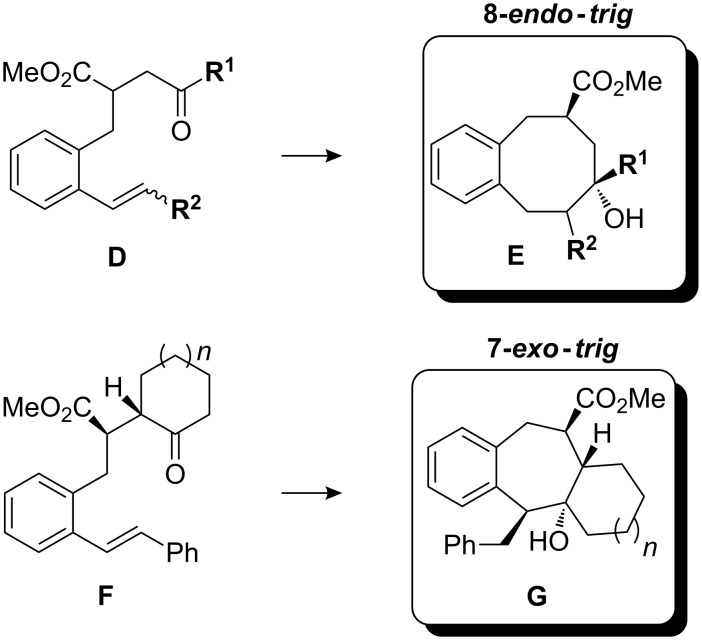
Typical products of samarium diiodide-induced 8-*endo*-*trig* cyclizations of β-styryl-substituted γ-ketoesters **D** and of 7-*exo*-*trig* cyclizations of β-styryl-substituted γ-ketoesters **F**.

γ-Ketoesters with substituents at the β-styryl carbon such as **D** cyclized with similar efficiency, affording in most cases the 8-*endo*-*trig*-cyclization products **E**. Only cyclohexanone derived γ-ketoesters **F** preferred the 7-*exo*-*trig* over the 8-*endo*-*trig*-cyclization mode (Scheme 16).

A typical side reaction, which was observed for methyl ketones or cyclohexanone derivatives, was the ketyl-aryl coupling reaction leading to hexahydronaphthalene derivatives in low yields.

In conclusion, the intramolecular 8-*endo*-*trig* samarium ketyl-alkene coupling reaction is a flexible tool for the construction of highly substituted cyclooctanol derivatives. A careful design of the precursors following the rules determined in this study allows the synthesis of functionalized benzannulated cyclooctanol derivatives with a high degree of regio- and stereocontrol.

## Experimental

**General:** All reactions were carried out under argon in flame-dried flasks, and the components were added by syringe. All solvents were dried by standard methods. Thin layer chromatography (TLC) was carried out on commercial Polygram Sil G/UV_254_ or Polygram Alox N/UV_254_ (Macherey & Nagel). Column chromatography was performed with 70–230 mesh silica gel (Merck) or neutral aluminium oxide (activity grade III; Fluka or Merck). Unless stated otherwise, ^1^H NMR and ^13^C NMR spectra were determined with Bruker AC 200, AC 300, DRX 500, Avance III 700 or Jeol Eclipse 500 instruments in CDCl_3_ solution. The chemical shifts refer to TMS or to the CDCl_3_ signal (*δ*_H_ = 7.26 ppm. *δ*_C_ = 77.16 ppm). IR spectra were measured with a Nicolet 205 5SXC FTIR-interferometer, equipped with a DTGS-detector. Melting points are uncorrected and determined with a melting point microscope Büchi 510. MS and HRMS analyses were performed with Finnigan MAT 711 (EI = 80 eV, 8 kV), MAT 95 (EI = 70 eV), MAT CH7A (EI = 80 eV, 3 kV) and CH5DF (FAB = 80 eV, 3 kV) instruments. Elemental analyses were performed with Perkin-Elmer and Vario EL Elementar analytical equipment.

Preparation of all cyclization precursors analogously to known procedures is described in Supporting Information File 1.

### SmI_2_-induced cyclization

**General procedure A** [60–61]**:** Samarium metal (2.4 equiv) and 1,2-diiodoethane (2.2 equiv) were placed under a flow of argon in a flame-dried, two-necked round-bottomed flask containing a magnetic stirring bar and a septum inlet. THF (12 mL/mmol of 1,2-diiodoethane) was added and the mixture was vigorously stirred at rt for 2 h. HMPA (18 equiv) was added to this solution of SmI_2_ (2.2 equiv), and after 10 min of stirring, a solution of the substrate (1 equiv) and *t*-BuOH (2 equiv) in THF (40 mL/mmol of substrate) was added over 2 h. The mixture was stirred at rt for 16 h and quenched with satd. aqueous NaHCO_3_ solution (20 mL/mmol of substrate). The phases were separated, and the aqueous layer was extracted with diethyl ether (3 × 15 mL/mmol of substrate). The combined organic layers were washed with water and brine (10 mL/mmol of substrate) and dried (Na_2_SO_4_).

**General procedure B** [62–63]**:** Samarium metal (2.48 g, 16.5 mmol) and iodine (3.81 g, 15.0 mmol) were placed under a flow of argon in a flame-dried, 250 mL round-bottomed flask containing a magnetic stirring bar. Then 150 mL of dry and oxygen-free THF was added and the mixture was covered from light, and stirred at rt for 24 h to give a 0.1 M solution of SmI_2_ in THF. This solution was stored in the dark under argon at rt and aliquots were transferred with a syringe (2.2 equiv) into the two-necked reaction flask, and the solution was then used as described in General procedure A.

#### SmI_2_-induced cyclization of **5a**

General procedure B: **5a** (0.300 g, 1.00 mmol), SmI_2_ (2.20 mmol), HMPA (3.16 mL, 18.0 mmol) and *t*-BuOH (0.148 g, 2.00 mmol). The crude product was purified by column chromatography (silica gel, ethyl acetate/hexane 1:9) to furnish **6** (0.126 g, 42%) as colourless crystals (mp 126–127 °C) and **5a** (0.069 g, 23%).

**Methyl (6*RS*,6a*SR*,11a*RS*)-11a-hydroxy-6,6a,7,8,9,10,11,11a,12,13-decahydro-5*H*-benzo[*a*]cyclohepta[*e*][8]annulene-6-carboxylate (6):** ^1^H NMR (CDCl_3_, 700 MHz): *δ* = 1.11–1.15, 1.20–1.33, 1.52–1.75, 1.77–1.81 (4 m, 2 H, 3 H, 6 H, 1 H, 6a-H, 7-H, 8-H, 9-H, 10-H, 11-H, OH), 1.88 (ddd, *J* = 2.2, 8.0, 14.4 Hz, 1 H, 12-H), 2.06 (ddd, *J* = 2.6, 11.6, 14.4 Hz, 1 H, 12-H), 2.68 (ddd, *J* = 2.6, 8.0, 14.0 Hz, 1 H, 13-H), 2.85 (dd, *J* = 4.6, 14.2 Hz, 1 H, 5-H), 3.01 (ddd, *J* = 4.6, 5.2, 10.6 Hz, 1 H, 6-H), 3.18 (ddd, *J* = 2.2, 11.6, 14.0 Hz, 1 H, 13-H), 3.62 (dd, *J* = 5.2, 14.2 Hz, 1 H, 5-H), 3.69 (s, 3 H, CO_2_Me), 6.89–6.91, 7.12–7.18 (2 m, 1 H, 3 H, Ar) ppm. ^13^C NMR (CDCl_3_, 175 MHz): *δ* = 23.6, 29.1, 29.4, 29.5 (4 t, C-7, C-8, C-9, C-10), 31.5 (t, C-13), 34.9 (t, C-5), 45.0 (d, C-6a), 46.9, 47.0 (2 t, C-11, C-12), 51.4 (d, C-6), 126.1, 127.0, 129.1, 130.5, 136.8, 142.0 (4 d, 2 s, Ar), 51.2, 176.1 (q, s, CO_2_Me) ppm. Signal for C-11a is not clearly visible. IR (KBr): 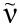 = 3500 (O–H), 3060–2855 (=C–H, C–H), 1715 (C=O) cm^–1^. C_19_H_26_O_3_ (302.4): calcd. C 75.46, H 8.67; found: C 75.36, H 8.78.

#### SmI_2_-induced cyclization of **7a**

General Procedure A: **7a** (0.296 g, 0.99 mmol), SmI_2_ (2.20 mmol), HMPA (3.16 mL, 18.0 mmol) and *t*-BuOH (0.148 g, 2.00 mmol). The crude product was purified by column chromatography (silica gel, ethyl acetate/hexane 1:9) then HPLC (*iso*-propanol/hexane 7:93) to furnish **8** (0.187 g, 63%) as colourless crystals (mp 74–75 °C).

**Methyl (4a*SR*,5*RS*,11*RS*,12a*RS*)-12a-hydroxy-11-methyl-1,2,3,4,4a,5,6,11,12,12a-decahydro-dibenzo[*a*,*e*][8]annulene-5-carboxylate (8):** ^1^H NMR (CDCl_3_, 500 MHz): δ = 1.14–1.18 (m, 2 H, 3-H, 4-H), 1.28 (d, *J* = 7.0 Hz, 3 H, 11-Me), 1.30–1.34, 1.38–1.46, 1.54–1.58, 1.63–1.70 (4 m, 1 H, 3 H, 1 H, 2 H, 1-H, 2-H, 3-H, 4-H, 4a-H), 1.96 (dd, *J* = 4.9, 15.2 Hz, 1 H, 12-H), 2.08 (dd, *J* = 11.5, 15.2 Hz, 1 H, 12-H), 2.63 (ddd, *J* = 4.7, 5.8, 10.6 Hz, 1 H, 5-H), 3.13–3.20 (m, 2 H, 6-H), 3.35 (dqd, *J* = 4.9, 7.0, 11.5 Hz, 1 H, 11-H), 3.73 (s, 3 H, CO_2_Me), 6.98–7.00, 7.12–7.21 (2 m, 1 H, 3 H, Ar) ppm, the signal for the OH group could not be assigned unambiguously. ^13^C NMR (CDCl_3_, 125 MHz): δ = 21.3 (t, C-2), 24.3 (q, 11-Me), 24.9 (t, C-3), 29.6 (t, C-4), 33.7 (d, C-11), 34.1 (t, C-6), 44.3 (t, C-1), 45.0 (d, C-4a), 53.2 (d, C-5), 54.0 (t, C-12), 73.2 (s, C-12a), 126.4, 127.4, 127.6, 131.0, 137.3, 144.8 (4 d, 2 s, Ar), 51.7, 176.6 (q, s, CO_2_Me) ppm. IR (KBr): 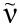 = 3510 (br, O-H), 3060–2860 (=C-H, C-H), 1725 (C=O) cm^−1^. MS (EI, 70 eV): *m/z* (%) = 302 (21) [M]^+^, 284 (100), 224 (64), 169 (57), 131 (53), 117 (60), 91 (44), 43 (46). C_19_H_26_O_3_ (302.4): calcd. C 75.46, H 8.67; found: C 75.23, H 8.71.

#### SmI_2_-induced cyclization of **4**

General Procedure A: **4** (0.200 g, 0.77 mmol), SmI_2_ (1.70 mmol), HMPA (2.43 mL, 13.8 mmol) and *t*-BuOH (0.114 g, 1.54 mmol). The crude product was purified by column chromatography (silica gel, ethyl acetate/hexane 1:9) to furnish **9** (0.080 g, 40%) as a colourless oil and **10** (0.067 g, 38%) as colourless crystals (mp 130–132 °C).

**Methyl (6*RS*,8*RS*,10*RS*)-8-hydroxy-8,10-dimethyl-5,6,7,8,9,10-hexahydro-benzo-[8]annulene-6-carboxylate (9):** Compound **9** shows temperature-dependent NMR spectra. At rt some signals appear broad; however, for measurements at 55 °C, the signals are more clearly seen. ^1^H NMR (CDCl_3_, 500 MHz, 55 °C): δ = 1.20 (s, 3 H, 8-Me), 1.33 (dd, *J* = 11.5, 14.7 Hz, 1 H, 7-H), 1.33 (d, *J* = 7.1 Hz, 3 H, 10-Me), 1.62 (dd, *J* = 11.4, 14.3 Hz, 1 H, 9-H), 1.67 (dd, *J* = 3.8, 14.7 Hz, 1 H, 7-H), 1.79–1.83 (m, 1 H, 9-H), 3.10 (dddd, *J* = 2.9, 3.8, 7.5, 11.5 Hz, 1 H, 6-H), 3.16 (dd, *J* = 2.9, 13.9 Hz, 1 H, 5-H), 3.36–3.43 (m, 1 H, 10-H), 3.43 (dd, *J* = 7.5, 13.9 Hz, 1 H, 5-H), 3.68 (s, 3 H, CO_2_Me), 6.97–6.99, 7.06–7.10, 7.17–7.25 (3 m, 1 H, 1 H, 2 H, Ar) ppm, the signal for the OH group could not be assigned unambiguously. ^13^C NMR (CDCl_3_, 125 MHz, 55 °C): δ = 23.2 (q, 10-Me), 30.2 (d, C-10), 32.3 (t, C-5), 36.0 (q, 8-Me), 36.5 (t, C-7), 42.2 (d, C-6), 55.1 (t, C-9), 71.5 (s, C-8), 125.1, 125.9, 127.1, 129.6, 136.5, 146.3 (4 d, 2 s, Ar), 51.7, 176.1 (q, s, CO_2_Me) ppm. IR (neat): 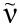 = 3500 (br, O-H), 3100–2840 (=C-H, C-H), 1715 (C=O) cm^–1^. HRMS (ESI) calcd for C_16_H_22_O_3_: [M+H]^+^ = 217.1255, [M+Na]^+^ = 239.1074, [M+K]^+^ = 255.0813; found: 217.1267, 239.1095, 255.0844.

**(2*RS*,5*SR*,7*SR*)-5,7-Dimethyl-1,5,6,7-tetrahydro-2,5-methano-4-benzoxonin-3(2*H*)-one (10): **^1^H NMR (CDCl_3_, 500 MHz): δ = 1.34 (d, *J* = 6.9 Hz, 3 H, 7-Me), 1.36 (s, 3 H, 5-Me), 1.43 (dd, *J* = 1.1, 13.9 Hz, 1 H, 12-H), 1.55 (dd, *J* = 11.3, 14.6 Hz, 1 H, 6-H), 1.74–1.79 (m, 1 H, 12-H), 2.05–2.09 (m, 1 H, 6-H), 2.76 (dqd, *J* = 1.3, 6.9, 11.3 Hz, 1 H, 7-H), 3.15 (dddd, *J* = 1.1, 2.5, 10.3, 12.8 Hz, 1 H, 2-H), 3.20 (dd, *J* = 12.8, 14.5 Hz, 1 H, 1-H), 3.31 (dd, *J* = 2.5, 14.5 Hz, 1 H, 1-H), 7.00–7.02, 7.15–7.18, 7.28–7.36 (3 m, 1 H, 1 H, 2 H, Ar) ppm. ^13^C NMR (CDCl_3_, 125 MHz): δ = 22.5 (q, 7-Me), 29.6 (q, 5-Me), 30.0 (d, C-7), 34.2 (t, C-12), 34.5 (t, C-1), 39.3 (d, C-2), 51.4 (t, C-6), 86.3 (s, C-5), 125.4, 126.7, 127.9, 130.4, 136.8, 146.2 (4 d, 2 s, Ar), 181.5 (s, C-3) ppm. IR (KBr): 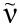 = 3065–2825 (=C-H, C-H), 1755 (C=O) cm^–1^. C_15_H_18_O_2_ (230.3): calcd. C 78.23, H 7.88; found: C 78.49, H 7.93.

#### SmI_2_-induced cyclization of **11**

General procedure A: Compound **11** (0.290 g, 1.01 mmol), SmI_2_ (2.22 mmol), HMPA (3.16 mL, 18.0 mmol) and *t*-BuOH (0.150 g, 2.02 mmol). The crude product was purified by column chromatography (silica gel, ethyl acetate/hexane 1:9) to furnish **12** (0.157 g, 54%) as a colourless oil and **13** (0.072 g, 28%) as colourless crystals (mp 117–119 °C).

**Methyl (6*RS*,8*RS*,10*SR*)-8-hydroxy-8-isopropyl-10-methyl-5,6,7,8,9,10-hexahydro-benzo[8]annulene-6-carboxylate (12):** Compound **12** shows temperature-dependent NMR spectra. At rt most signals appear very broad; however, for measurements at 50 °C, the signals are clearly seen. ^1^H NMR (CDCl_3_, 500 MHz, 50 °C): δ = 0.85, 0.86 (2 d, *J* = 6.9 Hz, 2 × 3 H, CH*Me*_2_), 1.08 (dd, *J* = 12.5, 14.7 Hz, 1 H, 7-H), 1.23 (br. s, 1 H, OH), 1.35 (d, *J* = 7.0 Hz, 3 H, 10-Me), 1.55 (sept, *J* = 6.9 Hz, 1 H, C*H*Me_2_), 1.62–1.69, 3.07–3.13 (2 m, 3 H, 2 H, 5-H, 6-H, 7-H, 9-H), 3.45 (dqd, *J* = 3.1, 7.0, 9.9 Hz, 1 H, 10-H), 3.54 (dd, *J* = 7.6, 14.0 Hz, 1 H, 5-H), 3.71 (s, 3 H, CO_2_Me), 6.99–7.01, 7.09–7.12, 7.21–7.30 (3 m, 1 H, 1 H, 2 H, Ar) ppm. ^13^C NMR (CDCl_3_, 125 MHz, 50 °C): δ = 16.7, 17.2 (2 q, CH*Me*_2_), 23.3 (q, 10-Me), 29.1 (d, C-10), 31.9 (t, C-7), 32.7 (t, C-5), 42.2 (d, C-6), 43.2 (d, *C*HMe_2_), 50.3 (t, C-9), 74.8 (s, C-8), 124.7, 125.8, 127.1, 129.7, 136.6, 146.9 (4 d, 2 s, Ar), 51.6, 176.2 (q, s, CO_2_Me) ppm. IR (neat): 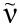 = 3515 (br, O-H), 3100–2845 (=C-H, C-H), 1720 (C=O) cm^–1^. MS (EI, 70 eV): *m/z* (%) = 290 (13) [M]^+^, 247 (70), 215 (85), 187 (54), 145 (73), 117 (40), 71 (26), 43 (100). C_18_H_26_O_3_ (290.4): calcd: C 74.45, H 9.02; found: C 74.71, H 8.83.

**(2*RS*,5*SR*,7*SR*)-5-Isopropyl-7-methyl-1,5,6,7-tetrahydro-2,5-methano-4-benzoxonin-3(2*H*)-one (13):** ^1^H NMR (CDCl_3_, 500 MHz): δ = 0.79, 0.89 (2 d, *J* = 6.9 Hz, 2 × 3 H, CH*Me*_2_), 1.27 (dd, *J* = 1.3, 13.9 Hz, 1 H, 12-H), 1.35 (d, *J* = 7.0 Hz, 3 H, 7-Me), 1.60 (dd, *J* = 11.1, 14.4 Hz, 1 H, 6-H), 1.75 (dd, *J* = 10.8, 13.9 Hz, 1 H, 12-H), 1.84 (sept, *J* = 6.9 Hz, 1 H, C*H*Me_2_), 1.95 (dd, *J* = 0.9, 14.4 Hz, 1 H, 6-H), 2.70–2.77 (m, 1 H, 7-H), 3.10–3.15 (m, 1 H, 2-H), 3.21 (dd, *J* = 12.8, 14.8 Hz, 1 H, 1-H), 3.32 (dd, *J* = 2.9, 14.8 Hz, 1 H, 1-H), 6.99–7.01, 7.14–7.17, 7.27–7.36 (3 m, 1 H, 1 H, 2 H, Ar) ppm. ^13^C NMR (CDCl_3_, 125 MHz): δ = 16.6, 17.4 (2 q, CH*Me*_2_), 22.9 (q, 7-Me), 29.6 (d, C-7), 30.5 (t, C-12), 34.6 (t, C-1), 38.5 (d, *C*HMe_2_), 38.9 (d, C-2), 45.3 (t, C-6), 91.2 (s, C-5), 125.4, 126.6, 127.9, 130.3, 136.7, 146.6 (4 d, 2 s, Ar), 181.7 (s, C-3) ppm. IR (KBr): 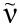 = 3075–2850 (=C-H, C-H), 1765 (C=O) cm^–1^. MS (EI = 70 eV): *m/z* (%) = 258 (46) [M]^+^, 215 (72), 187 (41), 173 (50), 145 (69), 117 (37), 91 (28), 71 (32), 43 (100). C_17_H_22_O_2_ (258.4): calcd: C 79.03, H 8.58; found: C 79.09, H 8.41.

#### SmI_2_-induced reaction of **14**

General procedure B: Compound **14** (0.276 g, 0.87 mmol), SmI_2_ (1.92 mmol), HMPA (2.75 mL, 15.7 mmol) and *t*-BuOH (0.129 g, 1.74 mmol). The crude product was purified by column chromatography (silica gel, ethyl acetate/hexane 1:9) to furnish **15** (0.103 g, 58%) as a colourless oil.

**Methyl 3-(2-isopropenylphenyl)propanoate (15): **^1^H NMR (CDCl_3_, 500 MHz): δ = 2.07 [br s, 3 H, =C(Ar)*Me*], 2.60 (t, *J* = 8.2 Hz, 2 H, 2-H), 2.98 (t, *J* = 8.2 Hz, 2 H, 3-H), 3.69 (s, 3 H, CO_2_Me), 4.87 (br. s, 1 H, =CH_2_), 5.22 (br. s, 1 H, =CH_2_), 7.10–7.20 (m, 4 H, Ar) ppm. ^13^C NMR (CDCl_3_, 100 MHz): δ = 25.3 [q, =C(Ar)*Me*], 28.3 (t, C-3), 35.9 (t, C-2), 115.3 (t, =CH_2_), 126.3, 127.2, 128.4, 129.0, 137.0, 143.9, 145.4 [4 d, 3 s, =*C*(*Ar*)Me], 51.7, 173.6 (q, s, CO_2_Me) ppm. IR (neat): 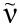 = 3065–2845 (=C-H, C-H), 1735 (C=O), 1640 (C=C) cm^–1^. MS (EI = 70 eV): *m/z* (%) = 204 (23) [M]^+^, 130 (47), 129 (100), 115 (42), 91 (29), 28 (47). HRMS (80 eV) calcd for C_13_H_16_O_2_: [M]^+^ = 204.11504; found: 204.11522.

#### SmI_2_-induced cyclization of **16**

General procedure A: Compound **16** (0.170 g, 0.49 mmol), SmI_2_ (1.08 mmol), HMPA (1.54 mL, 8.77 mmol) and *t*-BuOH (0.073 g, 0.98 mmol). The crude product was purified by column chromatography (silica gel, ethyl acetate/hexane 7:93 to 25:75) then HPLC (ethyl acetate/hexane 1:4) to furnish **17a** (0.058 g, 37%, mp 162–165 °C) and **17b** (0.035 g, 22%, mp 180–182 °C) as colourless crystals.

**(2*RS*,5*SR*,7*RS*)-5-Isopropyl-7-phenyl-1,5,6,7-tetrahydro-2,5-methano-4-benzoxonin-3(2*H*)-one (17a): **^1^H NMR (CDCl_3_, 500 MHz): δ = 0.89, 0.98 (2 d, *J* = 6.9 Hz, 2 × 3 H, CH*Me*_2_), 1.37 (dd, *J* = 1.3, 14.0 Hz, 1 H, 12-H), 1.84 (ddd, *J* = 1.3, 11.0, 14.0 Hz, 1 H, 12-H), 1.93 (sept, *J* = 6.9 Hz, 1 H, C*H*Me_2_), 2.13 (dd, *J* = 11.6, 14.0 Hz, 1 H, 6-H), 2.57 (td, *J* = 1.3, 14.0 Hz, 1 H, 6-H), 3.21 (dddd, *J* = 1.3, 3.3, 11.0, 13.0 Hz, 1 H, 2-H), 3.34 (dd, *J* = 13.0, 15.3 Hz, 1 H, 1-H), 3.55 (dd, *J* = 3.3, 15.3 Hz, 1 H, 1-H), 4.03 (dd, *J* = 1.3, 11.6 Hz, 1 H, 7-H), 6.93–6.96, 7.00–7.05, 7.08–7.13, 7.18–7.22, 7.27–7.32 (5 m, 1 H, 1 H, 2 H, 3 H, 2 H, Ar) ppm. ^13^C NMR (CDCl_3_, 125 MHz): δ = 16.7, 17.5 (2 q, CH*Me*_2_), 30.6 (d, C-7), 34.9 (t, C-12), 39.0 (t, C-1), 39.1 (d, *C*HMe_2_), 40.7 (d, C-2), 41.5 (t, C-6), 90.8 (s, C-5), 126.5, 126.7, 128.0, 128.3, 128.5, 128.6, 130.3, 136.4, 145.2, 145.8 (7 d, 3 s, Ar), 181.5 (s, C-3) ppm. IR (KBr): 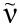 = 3085–2840 (=C-H, C-H), 1760 (C=O) cm^–1^. MS (EI, 70 eV): *m/z* (%) = 320 (75) [M]^+^, 309 (15), 277 (92), 179 (49), 91 (92), 43 (100). HRMS (80 eV) calcd for C_22_H_24_O_2_: [M]^+^ = 320.17764; found: 320.17735.

**(2*RS*,5*SR*,7*SR*)-5-Isopropyl-7-phenyl-1,5,6,7-tetrahydro-2,5-methano-4-benzoxonin-3(2*H*)-one (17b): **^1^H NMR (CDCl_3_, 500 MHz): δ = 0.93, 1.01 (2 d, *J* = 6.9 Hz, 2 × 3 H, CH*Me*_2_), 1.86 (sept, *J* = 6.9 Hz, 1 H, C*H*Me_2_), 2.34 (dd, *J* = 2.5, 14.5 Hz, 1 H, 6-H), 2.38 (dd, *J* = 9.5, 13.8 Hz, 1 H, 12-H), 2.44 (dd, *J* = 12.4, 14.5 Hz, 1 H, 6-H), 2.48 (dd, *J* = 1.0, 13.8 Hz, 1 H, 12-H), 3.29 (dddd, *J* = 1.0, 4.9, 9.5, 14.3 Hz, 1 H, 2-H), 3.30–3.36 (m, 2 H, 1-H), 4.40 (dd, *J* = 2.5, 12.4 Hz, 1 H, 7-H), 6.79–6.82, 7.04–7.07, 7.09–7.12, 7.23–7.29, 7.32–7.36 (5 m, 1 H, 2 H, 1 H, 3 H, 2 H, Ar) ppm. ^13^C NMR (CDCl_3_, 125 MHz): δ = 16.8, 17.3 (2 q, CH*Me*_2_), 37.0 (t, C-12), 37.9 (t, C-1), 39.5 (t, C-6), 40.3 (d, *C*HMe_2_), 42.5 (d, C-2), 42.8 (d, C-7), 90.6 (s, C-5), 126.2, 126.6, 128.0, 128.3, 128.3, 128.6, 132.0, 135.6, 144.3, 145.3 (7 d, 3 s, Ar), 176.2 (s, C-3) ppm. IR (KBr): 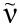 = 3065–2875 (=C-H, C-H), 1755 (C=O) cm^–1^. MS (EI, 70 eV): *m/z* (%) = 320 (100) [M]^+^, 277 (97), 179 (45), 91 (91), 43 (59). C_22_H_24_O_2_ (320.4): calcd: C 82.46, H 7.55; found: C 81.83, H 7.59.

#### SmI_2_-induced cyclization of **18**

General procedure A: Compound **18** (0.200 g, 0.77 mmol), SmI_2_ (1.70 mmol), HMPA (2.43 mL, 13.8 mmol) and *t*-BuOH (0.114 g, 1.54 mmol). The crude product was purified by column chromatography (silica gel, ethyl acetate/hexane 1:9) to furnish **19** (0.108 g, 54%) and **20** (0.040 g, 20%) as colourless oils.

**Methyl (6*RS*,8*SR*,9*SR*)-8-hydroxy-8,9-dimethyl-5,6,7,8,9,10-hexahydro-benzo[8]annulene-6-carboxylate (19):** Compound **19** shows temperature-dependent NMR spectra. At rt several signals appear broad; however, for measurements at 55 °C, signals are more clearly seen. ^1^H NMR (CDCl_3_, 500 MHz, 55 °C): δ = 0.97 (d, *J* = 7.0 Hz, 3 H, 9-Me), 1.17 (s, 3 H, 8-Me), 1.62 (dd, *J* = 2.8, 14.8 Hz, 1 H, 7-H), 1.77 (dd, *J* = 12.0, 14.8 Hz, 1 H, 7-H), 1.94–2.01 (m, 1 H, 9-H), 2.54 (dd, *J* = 9.1, 14.0 Hz, 1 H, 10-H), 2.96–3.08 (m, 3 H, 5-H, 6-H, 10-H), 3.22 (dd, *J* = 5.0, 13.8 Hz, 1 H, 5-H), 3.70 (s, 3 H, CO_2_Me), 7.07–7.15 (m, 4 H, Ar) ppm, the signal for the OH group could not be assigned unambiguously. ^13^C NMR (CDCl_3_, 125 MHz, 55 °C): δ = 17.0 (q, 9-Me), 32.0 (q, 8-Me), 35.1 (t, C-5), 35.8 (t, C-7), 37.4 (t, C-10), 43.0 (d, C-6), 47.0 (d, C-9), 74.8 (s, C-8), 126.8, 127.0, 130.6, 130.7, 138.1, 139.7 (4 d, 2 s, Ar), 51.7, 176.3 (q, s, CO_2_Me) ppm. IR (neat): 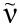 = 3510 (br, O-H), 3100–2850 (=C-H, C-H), 1720 (C=O) cm^−1^. C_16_H_22_O_3_ (262.3): calcd: C 73.25, H 8.45; found: C 73.43, H 8.30.

**Methyl (2*SR*,4*RS*,4a*SR*)-4-hydroxy-4-methyl-8-[(1*E*)-propen-1-yl]-1,2,3,4,4a,7-hexahydro-naphthalene-2-carboxylate (20): **^1^H NMR (CDCl_3_, 500 MHz): δ = 1.02 (s, 3 H, 4-Me), 1.79 (dd, *J* = 1.5, 6.6 Hz, 3 H, =CH*Me*), 1.87-1.91 (m, 2 H, 1-H, 3-H), 2.07 (dd, *J* = 3.8, 12.5 Hz, 1 H, 3-H), 2.37 (tt, *J* = 3.8, 12.9 Hz, 1 H, 2-H), 2.70–2.84 (m, 3 H, 1-H, 7-H), 3.21 (dd, *J* = 1.6, 3.8 Hz, 1 H, 4a-H), 3.72 (s, 3 H, CO_2_Me), 5.64 (qd, *J* = 6.6, 15.6 Hz, 1 H, =C*H*Me), 5.84–5.87, 5.89–5.92 (2 m, 2 × 1 H, 5-H, 6-H), 6.53 (qd, *J* = 1.5, 15.6 Hz, 1 H, C*H*=CHMe) ppm, the signal for the OH group could not be assigned unambiguously. ^13^C NMR (CDCl_3_, 125 MHz): δ = 18.9 (q, =CH*Me*), 22.0 (q, 4-Me), 27.5 (t, C-1), 31.2 (t, C-7), 41.0 (d, C-2), 44.0 (t, C-3), 50.2 (d, C-4a), 74.6 (s, C-4), 123.2, 124.8, 125.8, 126.6, 128.2, 129.3 (4 d, 2 s, =CH, =C_q_), 52.0, 175.2 (q, s, CO_2_Me) ppm. Due to instability of the sample and storage before the MS analysis was accomplished, only the rearomatized product could be detected. HRMS (ESI) calcd for C_16_H_20_O_3_: [M+Na]^+^ = 283.1305; found: 283.1295.

#### SmI_2_-induced cyclization of **21**

General procedure B: Compound **21** (0.270 g, 0.99 mmol), SmI_2_ (2.20 mmol), HMPA (3.16 mL, 18.0 mmol) and *t*-BuOH (0.148 g, 2.00 mmol). The crude product was purified by column chromatography (silica gel, ethyl acetate/hexane 1:9), then HPLC (*iso*-propanol/hexane 2:98) to furnish **22** (0.152 g, 56%) and **23** (0.012 g, 6%) as colourless oils.

**Methyl (6*RS*,8*SR*,9*SR*)-8-ethyl-8-hydroxy-9-methyl-5,6,7,8,9,10-hexahydro-benzo[8]annulene-6-carboxylate (22):** Compound **22** shows temperature-dependent NMR spectra. At rt several signals appear broad; however, for measurements at 40 °C, the signals are more clearly seen. ^1^H NMR (CDCl_3_, 500 MHz, 40 °C): δ = 0.87 (t, *J* = 7.2 Hz, 3 H, 8-CH_2_*Me*), 0.92 (d, *J* = 6.9 Hz, 3 H, 9-Me), 1.39, 1.50 (2 qd, *J* = 7.2, 14.4 Hz, 2 × 1 H, 8-C*H*_2_Me), 1.56–1.70 (m, 2 H, 7-H), 1.97–2.03 (m, 1 H, 9-H), 2.55 (dd, *J* = 8.5, 13.6 Hz, 1 H, 10-H), 2.97–3.02 (m, 1 H, 6-H), 3.05–3.13 (m, 2 H, 5-H, 10-H), 3.27 (dd, *J* = 4.0, 13.7 Hz, 1 H, 5-H), 3.72 (s, 3 H, CO_2_Me), 7.08–7.11, 7.14–7.18 (2 m, 1 H, 3 H, Ar) ppm, the OH group could not be assigned unambiguously. ^13^C NMR (CDCl_3_, 125 MHz, 40 °C): δ = 7.1 (q, 8-CH_2_*Me*), 16.0 (q, 9-Me), 33.4 (t, C-7), 35.3 (t, C-5), 36.6 (t, 8-*C*H_2_Me), 36.8 (t, C-10), 43.0 (d, C-6), 44.2 (d, C-9), 75.7 (s, C-8), 126.7, 126.9, 130.6, 130.7, 138.3, 139.7 (4 d, 2 s, Ar), 51.6, 176.4 (q, s, CO_2_Me) ppm. IR (neat): 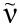 = 3535 (br, O-H), 3060–2860 (=C-H, C-H), 1720 (C=O) cm^–1^. HRMS (ESI) calcd for C_17_H_24_O_3_: [M+Na]^+^ = 299.1618; found: 299.1614.

**Methyl 3-{2-[(1*E*)-propen-1-yl]phenyl}propanoate (23):**^ 1^H NMR (CDCl_3_, 400 MHz): δ = 1.90 (dd, *J* = 1.6, 6.6 Hz, 3 H, =CH*Me*), 2.57 (t, *J* = 8.2 Hz, 2 H, 2-H), 2.99 (t, *J* = 8.2 Hz, 2 H, 3-H), 3.69 (s, 3 H, CO_2_Me), 6.12 (qd, *J* = 6.6, 15.5 Hz, 1 H, =C*H*Me), 6.62 (qd, *J* = 1.6, 15.5 Hz, 1 H, =C*H*Ar), 7.10–7.21, 7.39–7.42 (2 m, 3 H, 1 H, Ar) ppm. ^13^C NMR (CDCl_3_, 100 MHz): δ = 19.0 (q, =CH*Me*), 28.6 (t, C-3), 35.2 (t, C-2), 126.2, 126.8, 127.2, 128.1, 128.2, 129.3, 136.9, 137.2 (6 d, 2 s, ArCH=CH), 51.8, 173.6 (q, s, CO_2_Me) ppm. IR (neat): 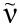 = 3065–2850 (=C-H, C-H), 1735 (C=O), 1600 (C=C) cm^–1^. HRMS (ESI) calcd for C_13_H_16_O_2_: [M+Na]^+^ = 227.1043, [M+K]^+^ = 243.0782; found: 227.1018, 243.0936.

#### SmI_2_-induced cyclization of **24**

General procedure A: Compound **24** (0.300 g, 1.04 mmol), SmI_2_ (2.30 mmol), HMPA (3.30 mL, 18.8 mmol) and *t*-BuOH (0.156 g, 2.10 mmol). The crude product was purified by column chromatography (silica gel, ethyl acetate/hexane 1:9) to furnish **25** (0.188 g, 63%) as colourless crystals (mp 89–90 °C).

**Methyl (6*RS*,8*RS*,9*SR*)-8-hydroxy-8-isopropyl-9-methyl-5,6,7,8,9,10-hexahydro-benzo[8]annulene-6-carboxylate (25):** Compound **25** shows temperature-dependent spectra. At rt most signals appear very broad; however, for measurements at 65 °C, the signals are clearly seen; ^1^H NMR (*d_6_*-DMSO, 500 MHz, 65 °C): δ = 0.78 (d, *J* = 7.0 Hz, 3 H, 9-Me), 0.85, 0.86 (2 d, *J* = 6.7 Hz, 2 × 3 H, CH*Me*_2_), 1.14 (dd, *J* = 12.7, 14.9 Hz, 1 H, 7-H), 1.40 (dd, *J* = 2.7, 14.9 Hz, 1 H, 7-H), 1.49 (sept, *J* = 6.7 Hz, 1 H, C*H*Me_2_), 2.01–2.07 (m, 1 H, 9-H), 2.33 (dd, *J* = 5.9, 13.8 Hz, 1 H, 10-H), 2.92 (dd, *J* = 2.4, 13.4 Hz, 1 H, 5-H), 2.99 (dddd, *J* = 2.4, 2.7, 6.6, 12.7 Hz, 1 H, 6-H), 3.33 (dd, *J* = 2.0, 13.8 Hz, 1 H, 10-H), 3.44 (dd, *J* = 6.6, 13.4 Hz, 1 H, 5-H), 3.61 (s, 3 H, CO_2_Me), 6.89–6.91, 7.06–7.12 (2 m, 1 H, 3 H, Ar) ppm, the signal for the OH group could not be assigned unambiguously. ^13^C NMR (*d_6_*-DMSO, 125 MHz, 65 °C): δ = 13.3 (q, 9-Me), 15.7, 16.2 (2 q, CH*Me*_2_), 28.0 (t, C-7), 33.2 (t, C-5), 34.9 (t, C-10), 36.0 (d, *C*HMe_2_), 39.9 (d, C-9), 40.9 (d, C-6), 74.8 (s, C-8), 125.2, 125.7, 129.5, 130.8, 137.4, 139.3 (4 d, 2 s, Ar), 50.8, 175.1 (q, s, CO_2_Me) ppm. IR (KBr): 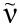 = 3490 (br, O-H), 3000–2840 (=C-H, C-H), 1715 (C=O) cm^–1^. MS (EI = 70 eV): *m/z* (%) = 290 (24) [M]^+^, 247 (74), 215 (90), 187 (100), 157 (54), 117 (54), 71 (21), 43 (80). C_18_H_26_O_3_ (290.4): calcd: C 74.45, H 9.02; found: C 74.51, H 8.74.

#### SmI_2_-induced cyclization of **26a**

General procedure A: Compound **26a** (0.300 g, 1.00 mmol), SmI_2_ (2.20 mmol), HMPA (3.16 mL, 18.0 mmol) and *t*-BuOH (0.148 g, 2.00 mmol). The crude product was purified by column chromatography (silica gel, ethyl acetate/hexane 1:9), then HPLC (ethyl acetate/hexane 15:85) to furnish **27** and **28** (0.054 g, 18%) as an inseparable mixture (**27**:**28** = 5:3 by integration of NMR signals) and **26a** (0.125 g, 42%) as colourless oils.

**Methyl (4a*SR*,5*RS*,12*SR*,12a*RS*)-12a-hydroxy-12-methyl-1,2,3,4,4a,5,6,11,12,-12a-decahydro-dibenzo[*a*,*e*][8]annulene-5-carboxylate (27): **^1^H NMR (CDCl_3_, 700 MHz): *δ* = 0.80–2.03 (m, 10 H, 1-H, 2-H, 3-H, 4-H, 4a-H, OH), 1.04 (d, *J* = 6.6 Hz, 3 H, 12-Me), 2.56–2.62 (m, 1 H, 12-H), 2.70 (dd, *J* = 11.3, 16.8 Hz, 1 H, 11-H), 2.82 (dd, *J* = 2.2, 15.0 Hz, 1 H, 6-H), 2.97 (dd, *J* = 3.8, 16.8 Hz, 1 H, 11-H), 3.00 (ddd, *J* = 2.2, 7.3, 9.6 Hz, 1 H, 5-H), 3.61 (dd, *J* = 7.3, 15.0 Hz, 1 H, 6-H), 3.63 (s, 3 H, CO_2_Me), 7.03–7.07, 7.10–7.15 (2 m, 2 × 2 H, Ar) ppm.

**Methyl (4a*S*,4b*R*,8a*R*,9*S*)-4b-hydroxy-1-[(1*E*)-prop-1-en-1-yl]-2,4a,4b,5,6,7,8,8a,-9,10-decahydro-phenanthrene-9-carboxylate (28):** ^1^H NMR (CDCl_3_, 700 MHz): *δ* = 0.80–2.03 (m, 15 H, 2-H, 5-H, 6-H, 7-H, 8-H, 8a-H, 9-H, 10-H, OH), 1.81 (dd, *J* = 1.2, 6.5 Hz, 3 H, =CH*Me*), 3.14 (dd, *J* = 3.8, 13.3 Hz, 1 H, 4a-H), 3.72 (s, 3 H, CO_2_Me), 5.66 (qd, *J* = 6.5, 15.4 Hz, 1 H, =C*H*Me), 5.83–5.85, 5.90–5.93 (2 m, 2 × 1 H, 3-H, 4-H), 6.53 (br. d, *J* = 15.4 Hz, 1 H, 1-C*H=*) ppm.

Mixture of **27** + **28**: ^13^C NMR (CDCl_3_, 175 MHz, 40 °C): δ = 17.3, 18.9, 20.1, 20.7, 21.2, 24.4, 25.2, 26.6, 27.4, 27.6, 30.5, 32.0, 37.3, 40.2, 41.5, 43.0, 44.2, 45.2, 45.3, 51.2, 51.5, 51.9, 75.4 (s, C-4b, **28**), 75.9 (s, C-12a, **27**), 123.0, 124.7, 126.0, 126.2, 128.0, 129.2 (4 d, 2 s, C-1, C-3, C-4, C-10a, *C*H=*C*HMe, **28**), 125.8, 126.7, 129.5, 132.6, 136.0, 140.4 (4 d, 2 s, Ar, **27**), 175.5 (s, C=O, **28**), 176.6 (s, C=O, **27**) ppm, no unambiguous assignment of signals was possible. IR (neat): 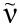 = 3510 (br, OH), 3060–2860 (=C-H, C-H), 1720 (br, C=O), 1600 (C=C) cm^–1^. HRMS (ESI) calcd for C_19_H_26_O_3_: [M+Na]^+^ = 325.1774; found: 325.1798.

#### SmI_2_-induced cyclization of **26b**

General procedure A: Compound **26b** (0.300 g, 1.00 mmol), SmI_2_ (2.20 mmol), HMPA (3.16 mL, 18.0 mmol) and *t*-BuOH (0.148 g, 2.00 mmol). The crude product was purified by column chromatography (silica gel, ethyl acetate/hexane 1:9) to furnish **29** (0.025 g, 9%) as colourless crystals (mp 119–121 °C), **23** (0.082 g, 27%) and **26b** (0.120 g, 40%) as colourless oils.

**(4a*RS*,5*RS*,12*RS*,12a*SR*)-5-Methyl-1,3,4,5,6,11,12,12a-octahydro-2*H*-4a,12-(epoxymethano)dibenzo[*a*,*e*][8]annulen-13-one (29): **^1^H NMR (CDCl_3_, 500 MHz): δ = 0.90 (d, *J* = 7.1 Hz, 3 H, 5-Me), 1.25–1.31, 1.39–1.48, 1.52–1.60, 1.65–1.81 (4 m, 1 H, 2 × 2 H, 3 H, 1-H, 2-H, 3-H, 4-H), 1.97–2.12 (m, 1 H, 12a-H), 2.19 (dqd, *J* = 2.0, 7.1, 7.2 Hz, 1 H, 12a-H), 2.57 (dd, *J* = 7.2, 14.6 Hz, 1 H, 6-H), 2.79 (br. d, *J* ≈ 14.6 Hz, 1 H, 6-H), 2.82 (ddd, *J* = 1.0, 5.2, 9.6 Hz, 1 H, 12-H), AB part of ABX system (δ_A_ = 3.24, δ_B_ = 3.27, *J*_AB_ = 15.2 Hz, *J*_AX_ = 9.6 Hz, *J*_BX_ = 5.2 Hz, 2 H, 11-H), 7.05–7.07, 7.14–7.21 (2 m, 1 H, 3 H, Ar) ppm. ^13^C NMR (CDCl_3_, 100 MHz): δ = 15.4 (q, 5-Me), 16.4, 16.5 (2 t, C-2, C-3), 28.6, 28.8 (2 t, C-1, C-4), 35.0 (t, C-6), 35.8 (t, C-11), 42.0 (d, C-5), 47.4 (d, C-12), 91.3 (s, C-4a), 126.7, 126.9, 131.3, 132.1, 137.7, 142.6 (4 d, 2 s, Ar) ppm, the signal for C-13 is not clearly visible. Signals for C-2, C-3, C-5, C-6, C-11, C-12, 5-Me, and three of the aromatic carbon atoms are broad. IR (KBr): 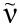 = 3045–2870 (=C-H, C-H), 1745 (C=O) cm^–1^. HRMS (ESI) calcd for C_18_H_22_O_2_: [M+H]^+^ = 271.1693, [M+Na]^+^ = 293.1512; found: 271.1692, 293.1509.

#### SmI_2_-induced cyclization of **30**

General procedure B: Compound **30** (0.288 g, 1.00 mmol), SmI_2_ (2.20 mmol), HMPA (3.16 mL, 18.0 mmol) and *t*-BuOH (0.148 g, 2.00 mmol). The crude product was purified by column chromatography (silica gel, ethyl acetate/hexane 1:9) to furnish **31** (0.022 g, 8%) as colourless crystals (mp 81–83 °C), **32** (0.064 g, 31%) and **30** (0.092 g, 32%) as colourless oils.

**Methyl (6*RS*,8*RS*,9*RS*)-8-hydroxy-8-isopropyl-9-methyl-5,6,7,8,9,10-hexahydro-benzo[8]annulene-6-carboxylate (31):** Compound **31** shows temperature-dependent spectra. At rt most signals appear very broad; for measurements at 55 °C, the signals are clearly seen. ^1^H NMR (CDCl_3_, 500 MHz, 55 °C): δ = 0.89, 0.90 (2 d, *J* = 6.8 Hz, 2 × 3 H, CH*Me*_2_), 0.94 (d, *J* = 7.0 Hz, 3 H, 9-Me), 1.49–1.65 (m, 1 H, 7-H), 1.62 (sept, *J* = 6.8 Hz, 1 H, C*H*Me_2_), 1.71–1.82 (m, 1 H, 7-H), 2.04–2.11 (m, 1 H, 9-H), 2.57 (dd, *J* = 8.4, 13.2 Hz, 1 H, 10-H), 2.87–2.97 (m, 1 H, 6-H), 3.07 (dd, *J* = 5.9, 13.7 Hz, 1 H, 5-H), 3.03–3.14 (m, 1 H, 10-H), 3.24–3.35 (m, 1 H, 5-H), 3.72 (s, 3 H, CO_2_Me), 7.07–7.09, 7.13–7.18 (2 m, 1 H, 3 H, Ar) ppm, the signal for the OH group could not be assigned unambiguously. ^13^C NMR (CDCl_3_, 125 MHz, 55 °C): δ = 15.4 (q, 9-Me), 16.3, 16.5 (2 q, CH*Me*_2_), 36.7 (d, *C*HMe_2_), 42.8 (d, C-9), 65.9 (s, C-8), 126.7, 127.0, 130.7, 130.8, 138.6, 139.9 (4 d, 2 s, Ar), 51.7, 176.5 (q, s, CO_2_Me) ppm. The signals for C-5, C-6 and C-7 are not clearly visible, the signals for C-9 and 9-Me are broad. IR (KBr): 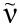 = 3490 (br, O-H), 3060–2850 (=C-H, C-H), 1710 (C=O) cm^–1^. HRMS (ESI) calcd for C_18_H_26_O_3_: [M+Na]^+^ = 313.1774; found: 313.1773.

**Methyl 3-{2-[(1*Z*)-propen-1-yl]phenyl}propanoate (32): **^1^H NMR (CDCl_3_, 250 MHz): δ = 1.72 (dd, *J* = 1.6, 7.0 Hz, 3 H, =CH*Me*), 2.54 (t, *J* = 8.2 Hz, 2 H, 2-H), 2.92 (t, *J* = 8.2 Hz, 2 H, 3-H), 3.66 (s, 3 H, CO_2_Me), 5.85 (qd, *J* = 7.0, 11.5 Hz, 1 H, =C*H*Me), 6.52 (qd, *J* = 1.6, 11.5 Hz, 1 H, =C*H*Ar), 7.18 (br. s, 4 H, Ar) ppm. ^13^C NMR (CDCl_3_, 125 MHz): δ = 14.4 (q, =CH*Me*), 28.8 (t, C-3), 34.8 (t, C-2), 126.1, 127.1, 127.8, 128.4, 129.0, 129.8, 136.4, 138.7 (6 d, 2 s, ArCH=CH), 51.7, 173.6 (q, s, CO_2_Me) ppm. IR (neat): 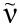 = 3065–2870 (=C-H, C-H), 1735 (C=O) cm^–1^. MS (EI, 70 eV): *m/z* (%) = 204 (56) [M]^+^, 144 (36), 129 (100), 115 (88), 91 (51), 77 (19), 18 (70); HRMS (80 eV) calcd for C_13_H_16_O_2_: [M]^+^ = 204.11504; found: 204.11464.

#### SmI_2_-induced cyclization of **33**

General procedure B: Compound **33** (0.314 g, 1.00 mmol), SmI_2_ (2.20 mmol), HMPA (3.16 mL, 18.0 mmol) and *t*-BuOH (0.148 g, 2.00 mmol). The crude product was purified by column chromatography (silica gel, ethyl acetate/hexane 1:9) to furnish **34** (0.075 g, 24%) as colourless crystals (mp 131–133 °C) and **35** (0.109 g, 47%) as a colourless oil.

**Methyl (6*RS*,8*RS*,9*RS*)-9-cyclopropyl-8-hydroxy-8-isopropyl-5,6,7,8,9,10-hexahydro-benzo[8]annulene-6-carboxylate (34):** Compound **35** shows temperature-dependent spectra. At rt most signals appear very broad; however, for measurements at 51 °C, the signals are clearly seen. ^1^H NMR (CDCl_3_, 500 MHz, 51 °C): δ = 0.27–0.32, 0.47–0.57, 0.62–0.69 (3 m, 1 H, 3 H, 1 H, 1'-H, 2'-H, 3'-H), 0.90, 0.98 (2 d, *J* = 6.7 Hz, 2 × 3 H, CH*Me*_2_), 1.12 (s, 1 H, OH), 1.28–1.33 (m, 1 H, 9-H), 1.60–1.68, 1.87–1.91 (2 m, 2 × 1 H, 7-H), 2.06 (sept, *J* = 6.7 Hz, 1 H, C*H*Me_2_), 2.68 (dd, *J* = 6.9, 13.9 Hz, 1 H, 10-H), 2.94–2.99 (m, 1 H, 6-H), 3.07 (dd, *J* = 4.1, 13.9 Hz, 1 H, 10-H), 3.18–3.21 (m, 1 H, 5-H), 3.41 (dd, *J* = 5.6, 13.7 Hz, 1 H, 5-H), 3.71 (s, 3 H, CO_2_Me), 7.04–7.07, 7.11–7.15, 7.26–7.30 (3 m, 1 H, 2 H, 1 H, Ar) ppm. ^13^C NMR (CDCl_3_, 125 MHz, 51 °C): δ = 3.8, 6.6 (2 t, C-2', C-3'), 11.7 (d, C-1'), 17.1, 17.3 (2 q, CH*Me*_2_), 34.8, 35.2, 35.9 (d, 2 t, *C*HMe_2_, C-5, C-10), 41.9 (d, C-6), 51.4 (d, C-9), 77.7 (s, C-8), 126.2, 126.5, 130.3, 131.0, 138.1, 140.2 (4 d, 2 s, Ar), 51.8, 176.5 (q, s, CO_2_Me) ppm, the signal for C-7 is not clearly visible, the signals for C-5, C-8, C-9, C-10, C-1', C-2', C-3', *C*HMe_2_, Ar (2 d, 2 s) are broad. IR (KBr): 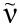 = 3490 (br, O-H), 3080–2875 (=C-H, C-H), 1715 (C=O) cm^–1^. C_20_H_28_O_3_ (316.4): calcd: C 75.91, H 8.92; found: C 76.09, H 8.88.

**Methyl 3-{2-[(*E*)-2-cyclopropylvinyl]phenyl}propanoate (35):**^1^H NMR (CDCl_3_, 500 MHz): δ = 0.82–0.91 (m, 4 H, 2'-H, 3'-H), 1.61 (ttd, *J* = 4.5, 8.5, 8.9 Hz, 1 H, 1'-H), 2.59 (t, *J* = 8.2 Hz, 2 H, 2-H), 3.02 (t, *J* = 8.2 Hz, 2 H, 3-H), 3.70 (s, 3 H, CO_2_Me), 5.60 (dd, *J* = 8.9, 15.5 Hz, 1 H, 1'-C*H*=), 6.69 (d, *J* = 15.5 Hz, 1 H, =C*H*Ar), 7.12–7.18, 7.36–7.39 (2 m, 3 H, 1 H, Ar) ppm. ^13^C NMR (CDCl_3_, 125 MHz): δ = 7.5 (t, C-2', C-3'), 15.0 (d, C-1'), 28.6 (t, C-2), 35.2 (t, C-3), 124.4 (d, =*C*HAr), 137.2 (d, 1'-*C*H=), 125.8, 126.8, 126.9, 129.3, 136.6, 137.0 (4 d, 2 s, Ar), 51.8, 173.6 (q, s, CO_2_Me) ppm. HRMS (ESI) calcd for C_15_H_18_O_2_: [M+Na]^+^ = 253.1199; found: 253.1201.

#### SmI_2_-induced cyclization of **36**

General procedure B: Compound **36** (0.200 g, 0.57 mmol), SmI_2_ (1.26 mmol), HMPA (1.80 mL, 10.3 mmol) and *t*-BuOH (0.085 g, 1.15 mmol). The crude product was purified by column chromatography (silica gel, ethyl acetate/hexane 1:9) to furnish **37** (0.094 g, 47%) as colourless crystals (mp 135–137 °C) and **36** (0.040 g, 20%) as a colourless oil.

**Methyl (6*RS*,8*RS*,9*RS*)-8-hydroxy-8-isopropyl-9-phenyl-5,6,7,8,9,10-hexahydro-benzo[8]annulene-6-carboxylate (37):**^1^H NMR (CDCl_3_, 500 MHz): δ = 1.07 (br. s, 1 H, OH), 1.10, 1.17 (2 d, *J* = 6.8 Hz, 2 × 3 H, CH*Me*_2_), 2.06 (dd, *J* = 12.4, 14.2 Hz, 1 H, 7-H), 2.07 (sept, *J* = 6.8 Hz, 1 H, C*H*Me_2_), 2.40–2.44 (m, 1 H, 7-H), 2.81 (dddd, *J* = 1.8, 2.6, 12.3, 12.4 Hz, 1 H, 6-H), 2.91 (dd, *J* = 3.0, 13.1 Hz, 1 H, 10-H), 3.02 (dd, *J* = 1.8, 14.8 Hz, 1 H, 5-H), 3.08 (dd, *J* = 11.9, 13.1 Hz, 1 H, 10-H), 3.21 (dd, *J* = 3.0, 11.9 Hz, 1 H, 9-H), 3.44 (dd, *J* = 12.3, 14.8 Hz, 1 H, 5-H), 3.74 (s, 3 H, CO_2_Me), 6.52–6.54, 6.75–6.77, 6.85–6.88, 7.04–7.14 (4 m, 1 H, 2 H, 1 H, 5 H, Ar) ppm. ^13^C NMR (CDCl_3_, 125 MHz): δ = 17.5, 17.7 (2 q, CH*Me*_2_), 35.2 (t, C-10), 35.3 (d, *C*HMe_2_), 38.3 (t, C-5), 38.5 (t, C-7), 40.5 (d, C-6), 57.8 (d, C-9), 74.2 (s, C-8), 125.9, 126.5, 127.5, 128.1, 128.9, 130.9, 134.4, 137.6, 139.4, 140.5 (7 d, 3 s, Ar), 52.1, 176.8 (q, s, CO_2_Me) ppm. IR (KBr): 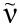 = 3485 (br, O-H), 3100–2845 (=C-H, C-H), 1710 (C=O) cm^–1^. MS (EI = 70 eV): *m/z* (%) = 352 (41) [M]^+^, 309 (15), 267 (22), 157 (70), 91 (100), 71 (40), 43 (95). C_23_H_28_O_3_ (352.5): calcd C 78.38, H 8.01; found: C 78.05, H 8.04.

#### SmI_2_-induced cyclization of **38a**

General procedure A: Compound **38a** (0.170 g, 0.47 mmol), SmI_2_ (1.04 mmol), HMPA (1.48 mL, 8.43 mmol) and *t*-BuOH (0.070 g, 0.94 mmol). The crude product was purified by column chromatography (silica gel, ethyl acetate/hexane 1:9), then HPLC (ethyl acetate/hexane 15:85) to furnish **39** (0.074 g, 44%) as colourless crystals (mp 114–115 °C).

**Methyl (4a*SR*,5*SR*,11*RS*,11a*SR*)-5-benzyl-4a-hydroxy-2,3,4,4a,5,10,11,11a-octahydro-1*H*-dibenzo[*a*,*d*][7]annulene-11-carboxylate (39):**^1^H NMR (CDCl_3_, 500 MHz): δ = 1.10 (s, 1 H, OH), 1.24–1.36, 1.42–1.51, 1.59–1.64, 1.66–1.75, 2.01–2.08 (5 m, 2 H, 2 × 1 H, 3 H, 1 H, 1-H, 2-H, 3-H, 4-H), 2.24 (ddd, *J* = 3.7, 11.2, 11.4 Hz, 1 H, 11a-H), 2.56 (ddd, *J* = 1.6, 11.2, 12.2 Hz, 1 H, 11-H), 2.75 (dd, *J* = 1.6, 15.0 Hz, 1 H, 10-H), 2.81 (dd, *J* = 3.3, 11.2 Hz, 1 H, 5-H), AB part of ABX system (δ_A_ = 3.11, δ_B_ = 3.19, *J*_AB_ = 13.3 Hz, *J*_AX_ = 3.3 Hz, *J*_BX_ = 11.2 Hz, 2 H, PhC*H*_2_), 3.57 (dd, *J* = 12.2, 15.0 Hz, 1 H, 10-H), 3.73 (s, 3 H, CO_2_Me), 6.54–6.56, 6.81–6.83, 6.87–6.91, 7.05–7.12 (4 m, 1 H, 2 H, 1 H, 5 H, Ar) ppm, the signal for the OH group could not be assigned unambiguously. ^13^C NMR (CDCl_3_, 125 MHz): δ = 21.9 (t, C-3), 25.8 (t, C-2), 28.5 (t, C-1), 35.7 (t, Ph*C*H_2_), 38.6 (t, C-10), 39.7 (t, C-4), 44.7 (d, C-11a), 47.8 (d, C-11), 64.9 (d, C-5), 72.7 (s, C-4a), 125.9, 126.7, 127.5, 128.1, 128.9, 130.6, 133.7, 138.1, 138.6, 141.0 (7 d, 3 s, Ar), 51.7, 176.6 (q, s, CO_2_Me) ppm. IR (KBr): 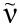 = 3430 (br, O-H), 3055–2845 (=C-H, C-H), 1705 (C=O) cm^–1^. MS (EI = 70 eV): *m/z* (%) = 364 (4) [M]^+^, 267 (39), 169 (39), 115 (23), 91 (100), 41 (10). HRMS (80 eV) calcd for C_24_H_28_O_3_: [M]^+^ = 364.2039; found: 364.2034.

#### SmI_2_-induced cyclization of **38b**

General procedure A: Compound **38b** (0.145 g, 0.40 mmol), SmI_2_ (0.88 mmol), HMPA (1.27 mL, 7.23 mmol) and *t*-BuOH (0.059 g, 0.80 mmol). The crude product was purified by column chromatography (silica gel, ethyl acetate/hexane 1:9) to furnish **40** (0.084 g, 63%) as colourless crystals (mp 193–195 °C).

**(4a*RS*,5*RS*,11*RS*,11a*RS*)-5-Benzyl-1,2,3,4,5,10,11,11a-octahydro-4a,11-(epoxy-methano)-dibenzo[*a*,*d*][7]annulen-12-one (40): **^1^H NMR (CDCl_3_, 500 MHz): δ = 1.11–1.29, 1.47–1.56, 1.71–1.75, 1.95–2.03, 2.19–2.23 (5 m, 2 H, 1 H, 2 × 2 H, 1 H, 1-H, 2-H, 3-H, 4-H), 2.54 (dd, *J* = 6.6, 11.4 Hz, 1 H, 11a-H), 2.65 (dd, *J* = 11.3, 12.8 Hz, 1 H, PhC*H*_2_), 2.71 (dd, *J* = 2.6, 6.6 Hz, 1 H, 11-H), 2.94 (dd, *J* = 2.4, 12.8 Hz, 1 H, PhC*H*_2_), 3.02 (dd, *J* = 2.4, 11.3 Hz, 1 H, 5-H), 3.23–3.31 (m, 2 H, 10-H), 6.29–6.31, 6.71–6.74, 6.81–6.85, 7.04–7.07, 7.11–7.15 (5 m, 1 H, 2 H, 2 × 1 H, 4 H, Ar) ppm. ^13^C NMR (CDCl_3_, 125 MHz): δ = 21.5 (t, C-3), 23.6 (t, C-2), 29.9 (t, C-1), 33.7 (t, C-4), 36.6 (t, C-10), 39.6 (t, Ph*C*H_2_), 43.5 (d, C-11a), 49.5 (d, C-5), 62.4 (d, C-11), 86.0 (s, C-4a), 126.3, 126.5, 127.1, 128.2, 129.2, 131.2, 133.3, 135.5, 137.6, 139.6 (7 d, 3 s, Ar), 178.2 (s, C-12) ppm. IR (neat): 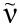 = 3060–2855 (=C-H, C-H), 1760 (C=O) cm^–1^. C_23_H_24_O_2_ (332.4): calcd: C 83.10, H 7.28; found: C 82.64, H 7.08.

#### SmI_2_-induced cyclization of **41**

General procedure A: Compound **41** (0.223 g, 0.81 mmol), SmI_2_ (1.80 mmol), HMPA (2.56 mL, 14.6 mmol) and *t*-BuOH (0.120 g, 1.62 mmol). The crude product was purified by column chromatography (silica gel, ethyl acetate/hexane 1:9), then HPLC (ethyl acetate/hexane 1:5) to furnish **42** (0.031 g, 14%) and **43** (0.014 g, 7%) as colourless oils.

**Methyl (2*SR*,4*RS*,4a*SR*)-4-hydroxy-4-methyl-8-(2-methylpropen-1-yl)-1,2,3,4,4a,7-hexahydro-naphthalene-2-carboxylate (42):**^1^H NMR (CDCl_3_, 500 MHz): δ = 1.08 (s, 3 H, 4-Me), 1.54, 1.74 (2 s, 2 × 3 H, =CMe_2_), 1.75–1.82 (m, 2 H, 1-H, 3-H), 2.04 (ddd, *J* = 1.8, 3.8, 12.5 Hz, 1 H, 3-H), 2.38 (tt, *J* = 3.8, 12.9 Hz, 1 H, 2-H), 2.50–2.62 (m, 2 H, 7-H), 2.71–2.77 (m, 2 H, 1-H, 4a-H), 3.67 (s, 3 H, CO_2_Me), 5.50 (br. s, 1 H, C*H*=CMe_2_), 5.82–5.88 (m, 2 H, 5-H, 6-H) ppm, the signal for the OH group could not be assigned unambiguously. ^13^C NMR (CDCl_3_, 125 MHz): δ = 19.5, 25.3 (2 q, =C*Me*_2_), 22.3 (q, 4-Me), 31.5 (t, C-7), 32.6 (t, C-1), 40.5 (d, C-2), 44.0 (t, C-3), 49.3 (d, C-4a), 74.2 (s, C-4), 123.7, 124.7, 125.9, 127.5, 128.8, 134.2 (3 d, 3 s, =CH, =C_q_), 51.9, 175.5 (q, s, CO_2_Me) ppm.

**(1*SR*,4*SR*,9a*SR*)-1-Methyl-6-(2-methylpropen-1-yl)-4,5,7,9a-tetrahydro-1,4-methano-2-benzoxepin-3(1*H*)-one (43**): ^1^H NMR (CDCl_3_, 500 MHz): δ = 1.51, 1.76 (2 s, 2 × 3 H, =CMe_2_), 1.54 (s, 3 H, 4-Me), AB part of ABX system (δ_A_ = 1.86, δ_B_ = 1.91, *J*_AB_ = 11.5 Hz, *J*_AX_ = 4.7 Hz, *J*_BX_ too small to see signal splitting, 2 H, 10-H), 2.22–2.27 (m, 1 H, 5-H), 2.52–2.67 (m, 3 H, 5-H, 7-H), 2.84–2.90 (m, 1 H, 4-H), 3.16–3.20 (m, 1 H, 9a-H), 5.49 (br. s, 1 H, C*H*=CMe_2_), 5.74–5.78, 5.84–5.88 (2 m, 2 × 1 H, 8-H, 9-H) ppm, the signal for the OH group could not be assigned unambiguously. ^13^C NMR (CDCl_3_, 125 MHz): δ = 19.7, 25.4 (2 q, =C*Me*_2_), 23.7 (q, 1-Me), 30.1 (t, C-5), 31.6 (t, C-7), 37.1, 37.9 (2 d, C-4, C-9a), 45.3 (t, C-10), 88.7 (s, C-1), 123.9, 124.2, 124.4, 126.8, 131.9, 136.1 (3 d, 3 s, =CH, =C_q_), 180.9 (s, C-3) ppm.

## Supporting Information

File 1 Experimental procedures and characterization data of synthesized compounds.
Supporting Information File 1 contains all experimental procedures for the syntheses of the starting materials **2**, **4**, **5**, **7**, **11**, **14**, **16**, **18**, **21**, **24**, **26**, **30**, **33**, **36**, **38**, and **41** and their analytical data.
