# Supplementary material for: Highly substituted benzannulated cyclooctanol derivatives by samarium diiodide-induced cyclizations
Source: Beilstein J Org Chem. 2010 Dec 28;6:1229–45. doi: 10.3762/bjoc.6.141 (PMC3028607; doi:10.3762/bjoc.6.141)
Supplement: File 1 — Experimental procedures and characterization data of synthesized compounds. Supporting Information File 1 contains all experimental procedures for the syntheses of the starting materials 2, 4, 5, 7, 11, 14, 16, 18, 21, 24, 26, 30, 33, 36, 38, and 41 and their analytical data. [file Beilstein_J_Org_Chem-06-1229-s001.pdf]

Supporting Information

for

Highly substituted benzannulated cyclooctanol derivatives by samarium diiodide-induced cyclizations

Jakub Saadi, Irene Brüdgam[‡] and Hans-Ulrich Reissig*

Address:

Freie Universität Berlin, Institut für Chemie und Biochemie, Takustrasse 3, D-14195 Berlin, Germany

Email: Hans-Ulrich Reissig - hreissig@chemie.fu-berlin.de

‡ Responsible for X-ray analyses

*Corresponding author

Experimental procedures and characterization data of synthesized compounds

General procedure 1 (GP1) for cyclopropanation of silyl enol ethers: To a refluxing mixture of silyl enol ether (1 equiv) and copper bis(acetylacetonate) (2.5 mol %) in ethyl acetate (~2 mL/mmol), a methyl diazoacetate (1.25-3 equiv) solution in ethyl acetate (0.15–0.3 mL/mmol) was added dropwise by a syringe pump over 2 h. Afterwards the heating was turned off and the mixture was allowed to cool over 1 h. It was concentrated and filtered through a short pad of neutral alumina (activity III) with hexane.

General procedure 2 (GP2) for alkylation of methyl 2-trimethylsiloxycyclopropane carboxylates: To a solution of 1.2 equiv of LDA (generated in situ from diisopropylamine and *n*-butyllithium in THF at –78 °C, 20 min) at –78 °C, 1 equiv of the corresponding cyclopropane derivative in THF was added. The reaction mixture was stirred for 2 h, then 1.2 equiv of 1-iodo-2-(iodomethyl)benzene (dissolved in THF) was added, and the mixture was stirred for 36 h at –78 °C. The mixture was quenched with satd. aqueous NH_4Cl solution and allowed to warm to room temperature. The two phases were separated, and the aqueous phase was repeatedly extracted with diethyl ether. The combined organic layers were washed with brine and dried (Na_2SO_4).

General procedure 3 (GP3) for triethylamine trihydrofluoride-induced cyclopropane ring cleavage of methyl 1-(2-iodobenzyl)-2-trimethylsiloxycyclo-propane carboxylates: The cyclopropane derivative (1 equiv) was dissolved in dry dichloromethane (4 mL/mmol) at rt. To this solution, triethylamine trihydrofluoride 1.5 equiv was added dropwise and the reaction mixture was stirred overnight. The mixture was diluted with dichloromethane, washed with water and dried (Na_2SO_4).

General procedure 4 (GP4) for palladium catalyzed cross-coupling reactions of aryl iodides: The aryl iodide (1 equiv) was dissolved under argon in the solvent (4 mL/mmol) in a pressure vessel. The palladium catalyst (2–5 mol %), the ligand (10–20 mol %), the base (1.5–3.5 equiv) and the coupling partner (1.2 equiv) were added and the vessel was sealed under argon. The mixture was heated to the desired temperature for 5–72 h. After cooling the mixture was diluted with ethyl acetate and

water. The two layers were separated and the aqueous phase was repeatedly extracted with ethyl acetate. The combined organic phase was washed with brine and dried (Na_2SO_4).

Methyl 2-ethyl-2-trimethylsiloxycyclopropane carboxylate (SP1): 2-Trimethylsiloxy-but-1-ene (4.00 g, 27.7 mmol), copper bis(acetylacetonate) (0.18 g, 0.68 mmol), ethyl acetate (60 mL), methyl diazoacetate (3.50 g, 35.0 mmol) and ethyl acetate (10 mL) were treated as described in **GP1**. The obtained yellowish oil was purified by reduced pressure distillation (bp 55-56 °C / 3 mbar) to give **SP1** (4.46 g, 74%) as a *cis*/*trans* mixture (60:40 calculated by integration of NMR signals) as a colourless oil. ^{1}H NMR ($CDCl_3$, 400 MHz): *trans*-isomer δ = 0.13 (s, 9 H, $SiMe_3$), 0.97 (t, *J* = 7.3 Hz, 3 H, CH_2*Me*), 1.49-1.88 (m, 5 H, 1-H, 3-H, CH_2Me), 3.66 (s, 3 H, CO_2Me); *cis*-isomer δ = 0.12 (s, 9 H, $SiMe_3$), 1.01 (t, *J* = 7.4 Hz, 3 H, CH_2*Me*), 1.49-1.88 (m, 5 H, 1-H, 3-H, CH_2Me), 3.65 (s, 3 H, CO_2Me) ppm. ^{13}C NMR ($CDCl_3$, 100 MHz): *trans*-isomer δ = 1.1 (q, $SiMe_3$), 10.2 (q, CH_2*Me*), 21.4 (t, C-3), 26.2 (d, C-1), 27.9 (t, *C*H_2Me), 65.3 (s, C-2), 51.8, 172.5 (q, s, CO_2Me); *cis*-isomer δ = 1.1 (q, $SiMe_3$), 10.0 (q, CH_2*Me*), 20.5 (t, C-3), 26.6 (d, C-1), 32.8 (t, *C*H_2Me), 64.7 (s, C-2), 51.6, 170.7 (q, s, CO_2Me) ppm. IR (neat): $\tilde{\nu}$ = 3090-2840 (=C-H, C-H), 1730 (C=O) cm^{-1}. HRMS (ESI) calcd for $C_{10}H_{20}O_3Si$: $[M+H]^+$ = 217.1255, $[M+Na]^+$ = 239.1074, $[M+K]^+$ = 255.0813; found: 217.1267, 239.1095, 255.0844.

Methyl 2-isopropyl-3,3-dimethyl-2-trimethylsiloxycyclopropane carboxylate (SP2): 3-Trimethylsiloxy-2,5-dimethylpent-2-ene (6.75 g, 36.3 mmol), copper bis(acetyl-acetonate) (0.24 g, 0.91 mmol), ethyl acetate (70 mL), methyl diazoacetate (9.10 g, 90.9 mmol), ethyl acetate (15 mL) were treated as described in **GP1**. The obtained yellowish oil was purified by reduced pressure distillation (bp 51-53 °C / 1.4 mbar) to give **SP2** (6.72 g, 72%) as a *cis*/*trans* mixture (58:42 calculated by integration of NMR signals) as a colourless oil. ^{1}H NMR ($CDCl_3$, 500 MHz): *trans*-isomer δ = 0.18 (s, 9 H, $SiMe_3$), 0.87, 0.95 (2 d, *J* = 6.8 Hz, 2 × 3 H, CH*Me*$_2$), 1.21, 1.31 (2 s, 2 × 3 H, 3-Me), 1.49 (s, 1 H, 1-H), 2.37 (sept, *J* = 6.8 Hz, 1 H, C*H*Me_2), 3.64 (s, 3 H, CO_2Me); *cis*-isomer δ = 0.16 (s, 9 H, $SiMe_3$), 0.99, 1.07 (2 d, *J* = 6.9 Hz, 2 × 3 H, CH*Me*$_2$), 1.20, 1.33 (2 s, 2

× 3 H, 3-Me), 1.50 (s, 1 H, 1-H), 2.18 (sept, *J* = 6.9 Hz, 1 H, C*H*Me_2), 3.62 (s, 3 H, CO_2Me) ppm. ^{13}C NMR ($CDCl_3$, 125 MHz): *trans*-isomer δ = 1.8 (q, $SiMe_3$), 16.1, 18.5, 19.4, 22.8 (4 q, 3-Me, CH*Me*$_2$), 27.2 (d, C-1), 31.7 (s, C-3), 37.0 (d, *C*HMe_2), 75.0 (s, C-2), 51.3, 171.4 (q, s, CO_2Me) ppm. The unreacted *trans* isomer was recovered by column chromatography (silica gel, hexane/ethyl acetate 90:10) from an unsuccessful alkylation reaction. This recovery allowed a detailed characterization of its ^{13}C NMR as well as correlation of the ^{1}H NMR signals for both of isomers from the mixture. The ^{13}C NMR spectrum of the mixture has not been taken. IR (neat): ṽ = 2955-2730 (=C-H, C-H), 1730 (C=O) cm^{-1}.

Methyl 1-(2-iodobenzyl)-2-methyl-2-trimethylsiloxycyclopropane carboxylate (2): Diisopropylamine (2.38 mL, 16.9 mmol), *n*-butyllithium (6.76 mL, 16.9 mmol), THF (20 mL), methyl 2-methyl-2-trimethylsiloxycyclopropanecarboxylate (**1**) (2.85 g, 14.1 mmol), THF (5 mL), 1-iodo-2-(iodomethyl)benzene (5.81 g, 16.9 mmol) and THF (10 mL) were treated as described in **GP2**. The obtained yellowish oil was purified by flash-chromatography (alumina, hexane → hexane/ethyl acetate = 90:10) to give **2** (3.26 g, 55%) as a colourless oil. ^{1}H NMR ($CDCl_3$, 500 MHz): δ = 0.23 (s, 9 H, $SiMe_3$), 0.94 (d, *J* = 6.0 Hz, 1 H, 3-H), 1.52 (s, 3 H, 2-Me), 1.67 (dd, *J* = 1.6, 6.0 Hz, 1 H, 3-H), 2.87 (d, *J* = 17.2 Hz, 1 H, ArC*H*$_2$), 3.56 (br. d, *J* = 17.2 Hz, 1 H, ArC*H*$_2$), 3.63 (s, 3 H, CO_2Me), 6.86-6.89, 7.23-7.27, 7.82-7.84 (3 m, 1 H, 2 H, 1 H, Ar) ppm. ^{13}C NMR ($CDCl_3$, 125 MHz): δ = 1.3 (q, $SiMe_3$), 21.2 (t, C-3), 25.6 (q, 2-Me), 34.8 (s, C-1), 40.2 (t, Ar*C*H_2), 63.0 (s, C-2), 102.0 (s, C-I), 127.7, 128.0, 128.3, 139.5, 143.0 (4 d, s, Ar), 52.2, 171.8 (q, s, CO_2Me) ppm. IR (neat): ṽ = 3060-2840 (=C-H, C-H), 1725 (C=O) cm^{-1}. $C_{16}H_{23}IO_3Si$ (418.3): calcd C 45.94, H 5.54; found C 45.65, H 5.28.

Methyl 2-ethyl-1-(2-iodobenzyl)-2-trimethylsilyloxycyclopropane carboxylate (SP4): Diisopropylamine (2.54 mL, 18.0 mmol), *n*-butyllithium (7.20 mL, 18.0 mmol), THF (25 mL), **SP1** (3.25 g, 15.0 mmol), THF (6 mL), 1-iodo-2-(iodomethyl)benzene (6.19 g, 18.0 mmol) and THF (12 mL) were treated as described in **GP2**. The obtained yellowish oil was purified by flash-chromatography (alumina, hexane → hexane/ethyl acetate = 90:10) to give **SP4** (5.52 g, 85%) as a colourless oil. ^{1}H NMR ($CDCl_3$,

500 MHz): δ = 0.21 (s, 9 H, $SiMe_3$), 0.92 (d, *J* = 6.0 Hz, 1 H, 3-H), 0.99 (t, *J* = 7.3 Hz, 3 H, CH_2*Me*), 1.67 (qd, *J* = 7.3, 14.5 Hz, 1 H, CH_2Me), 1.69 (d, *J* = 6.0 Hz, 1 H, 3-H), 1.81 (qd, *J* = 7.3, 14.5 Hz, 1 H, CH_2Me), 2.86 (d, *J* = 17.3 Hz, 1 H, ArCH_2), 3.57 (d, *J* = 17.3 Hz, 1 H, ArCH_2), 3.61 (s, 3 H, CO_2Me), 6.86-6.89, 7.23-7.27, 7.82-7.84 (3 m, 1 H, 2 H, 1 H, Ar) ppm. ^{13}C NMR ($CDCl_3$, 125 MHz): δ = 1.3 (q, $SiMe_3$), 10.0 (q, CH_2*Me*), 25.4 (t, C-3), 27.7 (t, *C*H_2Me), 34.3 (s, C-1), 40.3 (t, Ar*C*H_2), 67.8 (s, C-2), 102.0 (s, C-I), 127.7, 127.9, 128.3, 139.5, 143.1 (4 d, s, Ar), 52.2, 173.8 (q, s, CO_2Me) ppm. IR (neat): ṽ = 3060-2835 (=C-H, C-H), 1720 (C=O) cm^{-1}. $C_{17}H_{25}IO_3Si$ (432.4): calcd C 47.22, H 5.83; found C 47.18, H 5.85.

Methyl 7-(2-iodobenzyl)-1-trimethylsiloxybicyclo[4.1.0]heptane-7-carboxylate (SP5): Diisopropylamine (4.02 mL, 28.5 mmol), *n*-butyllithium (11.4 mL, 28.5 mmol), THF (30 mL), methyl 1-trimethylsiloxybicyclo[4.1.0]heptane-7-carboxylate (5.75 g, 23.7 mmol), THF (10 mL), 1-iodo-2-(iodomethyl)benzene (9.79 g, 28.5 mmol) and THF (15 mL) were treated as described in **GP2**. The obtained yellowish oil was purified by flash-chromatography (alumina, hexane → hexane/ethyl acetate = 90:10) to give **SP5** (10.7 g, 98%) as a colourless oil. ^{1}H NMR ($CDCl_3$, 500 MHz): δ = 0.24 (s, 9 H, $SiMe_3$), 1.12-1.32, 1.34-1.47, 1.94-2.03, 2.05-2.12 (4 m, 4 × 2 H, 2-H, 3-H, 4-H, 5-H), 1.30 (dd, *J* = 1.7, 8.7 Hz, 1 H, 6-H), 2.94 (d, *J* = 16.6 Hz, 1 H, ArCH_2), 3.35 (d, *J* = 16.6 Hz, 1 H, ArCH_2), 3.57 (s, 3 H, CO_2Me), 6.86-6.90, 7.27-7.31, 7.45-7.47, 7.80-7.82 (4 m, 4 × 1 H, Ar) ppm. ^{13}C NMR ($CDCl_3$, 125 MHz): δ = 1.5 (q, $SiMe_3$), 19.4, 21.0, 21.3, 30.6 (4 t, C-2, C-3, C-4, C-5), 30.3 (d, C-6), 36.8 (s, C-7), 43.7 (t, Ar*C*H_2), 62.2 (s, C-1), 101.6 (s, C-I), 127.9, 128.3, 129.1, 139.4, 142.5 (4 d, s, Ar), 51.6, 171.8 (q, s, CO_2Me) ppm. IR (neat): ṽ = 3060-2855 (=C-H, C-H), 1725 (C=O) cm^{-1}. $C_{19}H_{27}IO_3Si$ (458.4): calcd C 49.78, H 5.94; found C 49.74, H 5.92.

Methyl 8-(2-iodobenzyl)-1-trimethylsiloxybicyclo[5.1.0]octane-8-carboxylate (SP6): Diisopropylamine (2.00 mL, 14.1 mmol), *n*-butyllithium (5.62 mL, 14.1 mmol), THF (20 mL), methyl 1-trimethylsiloxybicyclo[5.1.0]octane-8-carboxylate (3.00 g, 11.7 mmol), THF (8 mL), 1-iodo-2-(iodomethyl)benzene (4.30 g, 12.5 mmol) and THF (10 mL) were treated as described in **GP2**. The obtained yellowish oil was purified by flash-

chromatography (alumina, hexane → hexane/ethyl acetate = 90:10) to give **SP6** (4.46 g, 81%) as a colourless oil. ^{1}H NMR ($CDCl_3$, 500 MHz): δ = 0.28 (s, 9 H, $SiMe_3$), 1.18-1.21 (m, 1 H, 7-H), 1.23-1.32, 1.35-2.44, 1.66-1.86, 1.89-1.93, 2.06-2.12 (5 m, 2 × 1 H, 4 H, 1 H, 3 H, 2-H, 3-H, 4-H, 5-H, 6-H), 2.72 (d, *J* = 17.2 Hz, 1 H, ArCH_2), 3.49 (d, *J* = 17.2 Hz, 1 H, ArCH_2), 3.58 (s, 3 H, CO_2Me), 6.87-6.90, 7.26-7.33, 7.81-7.83 (3 m, 1 H, 2 H, 1 H, Ar) ppm. ^{13}C NMR ($CDCl_3$, 125 MHz): δ = 1.4 (q, $SiMe_3$), 25.2, 25.8, 28.7, 31.8, 32.6 (5 t, C-2, C-3, C-4, C-5, C-6), 38.7 (d, C-7), 40.5 (s, C-8), 43.9 (t, ArCH_2), 70.0 (s, C-1), 102.1 (s, C-I), 127.7, 127.9, 128.4, 139.3, 142.8 (4 d, s, Ar), 51.7, 172.7 (q, s, CO_2Me) ppm. IR (neat): $\tilde{\nu}$ = 3070-2840 (=C-H, C-H), 1710 (C=O) cm^{-1}. $C_{20}H_{29}IO_3Si$ (472.4): calcd C 50.85, H 6.19; found C 50.92, H 6.11.

Methyl 2-(2-iodobenzyl)-4-oxohexanoate (SP7): SP4 (2.26 g, 5.23 mmol), dichloromethane (21 mL) and triethylamine trihydrofluoride (1.26 g, 7.82 mmol) were treated as described in **GP3**. The obtained yellowish oil was purified by column chromatography (silica gel, hexane/ethyl acetate = 90:10) to give **SP7** (1.38 g, 73%) as a colourless oil. ^{1}H NMR ($CDCl_3$, 500 MHz): δ = 1.02 (t, *J* = 7.3 Hz, 3 H, 6-H), 2.34-2.46 (m, 2 H, 5-H), 2.50 (dd, *J* = 4.1, 17.7 Hz, 1 H, 3-H), 2.88 (dd, *J* = 8.5, 13.6 Hz, 1 H, ArCH_2), 2.89 (dd, *J* = 9.2, 17.7 Hz, 1 H, 3-H), 3.13 (dd, *J* = 7.1, 13.6 Hz, 1 H, ArCH_2), 3.27 (dddd, *J* = 4.1, 7.1, 8.5, 9.2 Hz, 1 H, 2-H), 3.63 (s, 3 H, CO_2Me), 6.89-6.93, 7.14-7.16, 7.25-7.28, 7.81-7.83 (4 m, 4 × 1 H, Ar) ppm. ^{13}C NMR ($CDCl_3$, 125 MHz): δ = 7.7 (q, C-6), 36.0 (t, C-5), 40.7 (d, C-2), 42.2 (t, ArCH_2), 43.0 (t, C-3), 100.9 (s, C-I), 128.4, 128.6, 130.3, 139.9, 141.4 (4 d, s, Ar), 52.0, 175.0 (q, s, CO_2Me), 209.2 (s, C-4) ppm. IR (neat): $\tilde{\nu}$ = 3050-2850 (=C-H, C-H), 1730, 1710 (C=O) cm^{-1}. $C_{14}H_{17}IO_3$ (360.2): calcd C 46.68, H 4.76; found C 46.62, H 4.64.

Methyl (2*RS*)-3-(2-iodophenyl)-2-[(1*SR*)-2-oxocyclohexyl]propanoate (SP8a) and **methyl (2*RS*)-3-(2-iodophenyl)-2-[(1*RS*)-2-oxocyclohexyl]propanoate (SP8b): SP5** (10.7 g, 23.3 mmol), dichloromethane (90 mL) and triethylamine trihydrofluoride (5.64 g, 35.0 mmol) were treated as described in **GP3**. The obtained yellowish solid was purified by column chromatography (silica gel, hexane/ethyl acetate = 95:5) to give **SP8a** (2.35 g, 26%), **SP8b** (1.06 g, 12%) and a mixture of both diastereomers **SP8a/b** (5.38 g, 60%)

as colourless crystals (**SP8a**: mp 59-60 °C, **SP8b**: mp 96-98 °C). **SP8a**: ^{1}H NMR ($CDCl_3$, 500 MHz): δ = 1.62-1.79 (m, 3 H, 4'-H, 5'-H, 6'-H), 1.89-1.93, 2.03-2.06 (2 m, 2 × 1 H, 4'-H, 5'-H), 2.09-2.13 (m, 1 H, 6'-H), 2.31-2.37 (m, 1 H, 3'-H), 2.47-2.51 (m, 1 H, 3'-H), 2.61-2.66 (m, 1 H, 1'-H), 2.94-3.02 (m, 2 H, 3-H), 3.16 (td, *J* = 7.2, 8.4 Hz, 1 H, 2-H), 3.53 (s, 3 H, CO_2Me), 6.87-6.90, 7.20-7.26, 7.79-7.81 (3 m, 1 H, 2 H, 1 H, Ar) ppm. ^{13}C NMR ($CDCl_3$, 125 MHz): δ = 24.8, 27.7 (2 t, C-4', C-5'), 31.0 (t, C-6'), 40.9 (t, C-3), 42.2 (t, C-3'), 45.2 (d, C-2), 52.4 (d, C-1'), 100.7 (s, C-I), 128.3, 128.4, 130.1, 139.8, 141.9 (4 d, s, Ar), 51.5, 174.2 (q, s, CO_2Me), 210.8 (s, C-2') ppm. IR (KBr): $\tilde{\nu}$ = 3070-2855 (=C-H, C-H), 1725, 1710 (C=O) cm^{-1}. MS (EI = 70 eV): *m/z* (%) = 386 (14) $[M]^+$, 289 (17), 217 (12), 98 (100), 90 (12), 70 (10). $C_{16}H_{19}IO_3$ (386.2): calcd C 49.76, H 4.96; found C 49.89, H 4.82.

SP8b: ^{1}H NMR ($CDCl_3$, 500 MHz): δ = 1.59-1.77 (m, 3 H, 4'-H, 5'-H, 6'-H), 1.96-2.01, 2.08-2.13 (2 m, 2 × 1 H, 4'-H, 5'-H), 2.32-2.44 (m, 3 H, 3'-H, 6'-H), 2.79 (dd, *J* = 11.1, 13.2 Hz, 1 H, 3-H), 2.82-2.86 (m, 1 H, 1'-H), 2.95 (ddd, *J* = 4.7, 8.6, 11.1 Hz, 1 H, 2-H), 3.06 (dd, *J* = 4.7, 13.2 Hz, 1 H, 3-H), 3.43 (s, 3 H, CO_2Me), 6.88-6.92, 7.09-7.11, 7.21-7.25, 7.80-7.82 (4 m, 4 × 1 H, Ar) ppm. ^{13}C NMR ($CDCl_3$, 125 MHz): δ = 25.4, 27.7 (2 t, C-4', C-5'), 31.4 (t, C-6'), 40.9 (t, C-3), 42.2 (t, C-3'), 45.4 (d, C-2), 53.1 (d, C-1'), 100.6 (s, C-I), 128.2, 128.5, 130.2, 139.7, 141.7 (4 d, s, Ar), 51.4, 175.5 (q, s, CO_2Me), 211.1 (s, C-2') ppm. IR (KBr): $\tilde{\nu}$ = 3075-2855 (=C-H, C-H), 1725, 1700 (C=O) cm^{-1}. MS (EI = 70 eV): *m/z* (%) = 386 (13) $[M]^+$, 289 (18), 259 (13), 217 (13), 98 (100), 90 (11), 55 (12). $C_{16}H_{19}IO_3$ (386.2): calcd C 49.76, H 4.96; found C 49.77, H 5.01.

Methyl (2*RS*)-3-(2-iodophenyl)-2-[(1*SR*)-2-oxocycloheptyl]propanoate (SP9a) and **methyl (2*RS*)-3-(2-iodophenyl)-2-[(1*RS*)-2-oxocycloheptyl]propanoate (SP9b): SP6** (4.30 g, 9.10 mmol), dichloromethane (100 mL) and triethylamine trihydrofluoride (2.20 g, 13.7 mmol) were treated as described in **GP3**. The obtained yellowish solid was purified by column chromatography (silica gel, hexane/ethyl acetate = 90:10 → 85:15) to give **SP9a** (1.33 g, 37%) as colourless crystals (mp 57-58 °C), **SP9b** (0.64 g, 18%) and a mixture of both diastereomers **SP9a/b** (1.35 g, 37%) as colourless oils. **SP9a**: ^{1}H NMR ($CDCl_3$, 500 MHz): δ = 1.31-1.36, 1.42-1.50, 1.53-1.61, 1.84-1.98 (4 m, 2 H, 2 × 1 H, 4 H, 4'-H, 5'-H, 6'-H, 7'-H), 2.48-2.53 (m, 1 H, 3'-H), 2.59 (ddd, *J* = 3.6, 11.8, 14.2 Hz,

1 H, 3'-H), 2.74 (ddd, J = 3.4, 7.8, 11.1 Hz, 1 H, 1'-H), 2.90 (dd, J = 5.8, 13.7 Hz, 1 H, 3-H), 3.01 (dd, J = 9.7, 13.7 Hz, 1 H, 3-H), 3.29 (ddd, J = 5.8, 7.8, 9.7 Hz, 1 H, 2-H), 3.54 (s, 3 H, CO_2Me), 6.87-6.90, 7.16-7.25, 7.79-7.81 (3 m, 1 H, 2 H, 1 H, Ar) ppm. ^{13}C NMR ($CDCl_3$, 125 MHz): δ = 24.6, 28.4, 28.8, 29.6, (4 t, C-4', C-5', C-6', C-7'), 41.0 (t, C-3), 43.4 (t, C-3'), 47.8 (d, C-2), 54.0 (d, C-1'), 100.7 (s, C-I), 128.3, 128.5, 130.3, 139.8, 141.7 (4 d, s, Ar), 51.6, 173.9 (q, s, CO_2Me), 213.8 (s, C-2') ppm. IR (KBr): $\tilde{\nu}$ = 3055-2855 (=C-H, C-H), 1730, 1700 (C=O) cm^{-1}. $C_{17}H_{21}IO_3$ (400.3): calcd C 51.01, H 5.29; found C 51.15, H 5.18.

SP9b: ^1H NMR ($CDCl_3$, 500 MHz): δ = 1.26-1.36, 1.43-1.55, 1.65-1.73, 1.85-1.94, 2.08-2.12 (5 m, 1 H, 2 H, 1 H, 3 H, 1 H, 4'-H, 5'-H, 6'-H, 7'-H), 2.45 (ddd, J = 4.1, 11.5, 15.6 Hz, 1 H, 3'-H), 2.56 (ddd, J = 4.5, 4.6, 15.6 Hz, 1 H, 3'-H), 2.90 (dd, J = 10.2, 13.2 Hz, 1 H, 3-H), 2.97 (ddd, J = 3.0, 8.3, 10.1 Hz, 1 H, 1'-H), 3.03 (ddd, J = 4.5, 8.3, 10.2 Hz, 1 H, 2-H), 3.11 (dd, J = 4.5, 13.2 Hz, 1 H, 3-H), 3.44 (s, 3 H, CO_2Me), 6.88-6.91, 7.13-7.25, 7.80-7.82 (3 m, 1 H, 2 H, 1 H, Ar) ppm. ^{13}C NMR ($CDCl_3$, 125 MHz): δ = 24.0, 29.0, 29.1, 29.2 (4 t, C-4', C-5', C-6', C-7'), 41.3 (t, C-3), 43.4 (t, C-3'), 47.7 (d, C-2), 54.0 (d, C-1'), 100.6 (s, C-I), 128.2, 128.5, 130.4, 139.8, 141.6 (4 d, s, Ar), 51.5, 175.2 (q, s, CO_2Me), 214.5 (s, C-2') ppm. IR (neat): $\tilde{\nu}$ = 3075-2850 (=C-H, C-H), 1720, 1690 (C=O) cm^{-1}. $C_{17}H_{21}IO_3$ (400.3): calcd C 51.01, H 5.29; found C 50.99, H 5.06.

Methyl 3,3,5-trimethyl-4-oxohexanoate (SP10): SP2 (1.30 g, 5.03 mmol), dichloromethane (20 mL) and triethylamine trihydrofluoride (1.21 g, 7.51 mmol) were treated as described in **GP3**. The obtained yellowish oil was purified by column chromatography (silica gel, hexane/ethyl acetate = 90:10) to give **SP10** (0.85 g, 91%) as a colourless oil. ^1H NMR ($CDCl_3$, 270 MHz): δ = 1.06 (d, J = 6.7 Hz, 6 H, 5-Me, 6-H), 1.25 (s, 6 H, 3-Me), 2.54 (s, 2 H, 2-H), 3.10 (sept, J = 6.7 Hz, 1 H, 5-H), 3.61 (s, 3 H, CO_2Me) ppm. ^{13}C NMR ($CDCl_3$, 67 MHz): δ = 20.0 (q, 5-Me, C-6), 24.8 (q, 3-Me), 34.6 (d, C-5), 43.8 (t, C-2), 46.4 (s, C-3), 51.4, 172.1 (q, s, CO_2Me) 217.5 (s, C-4) ppm.

Methyl 2-(2-iodobenzyl)-3,3,5-trimethyl-4-oxohexanoate (SP11): Diisopropylamine (300 µL, 2.48 mmol), *n*-butyllithium (850 µL, 2.13 mmol), THF (4 mL), **SP10** (351 mg, 1.89 mmol), THF (2 mL), 1-iodo-2-(iodomethyl)benzene (776 mg, 2.26 mmol) and THF

(2 mL) were treated in analogy to **GP2**. The obtained yellowish oil was purified by column chromatography (silica gel, hexane/ethyl acetate = 90:10) to give **SP11** (592 mg, 78%) as a colourless oil. ^{1}H NMR ($CDCl_3$, 500 MHz): δ = 1.07, 1.08 (2 d, *J* = 6.8 Hz, 2 × 3 H, 5-Me, 6-H), 1.31, 1.42 (2 s, 2 × 3 H, 3-Me), 2.83 (dd, *J* = 3.3, 13.4 Hz, 1 H, ArC*H*$_2$), 2.97 (dd, *J* = 12.3, 13.4 Hz, 1 H, ArC*H*$_2$), 3.16 (sept, *J* = 6.8 Hz, 1 H, 5-H), 3.21 (dd, *J* = 3.3, 12.3 Hz, 1 H, 2-H), 3.43 (s, 3 H, CO_2Me), 6.86-6.89, 7.17-7.24, 7.78-7.80 (3 m, 1 H, 2 H, 1 H, Ar) ppm. ^{13}C NMR ($CDCl_3$, 125 MHz): δ = 20.1, 20.5 (2 q, 5-Me, C-6), 21.9, 22.3 (2 q, 3-Me), 34.8 (d, C-5), 38.8 (t, Ar*C*H$_2$), 50.6 (d, C-2), 51.2 (s, C-3), 100.7 (s, C-I), 128.3, 128.4, 130.3, 139.8, 141.8 (4 d, s, Ar), 51.2, 174.1 (q, s, CO_2Me), 218.1 (s, C-4) ppm. IR (neat): ṽ = 3055-2840 (=C-H, C-H), 1730, 1700 (C=O) cm^{-1}. $C_{17}H_{23}IO_3$ (402.3): calcd C 50.76, H 5.76; found C 50.81, H 5.86.

Methyl 2-(2-isopropenylbenzyl)-4-oxopentanoate (4): Methyl 2-(2-iodobenzyl)-4-oxopentanoate (400 mg, 1.16 mmol), palladium(II) acetate (13 mg, 0.058 mmol), triphenylphosphine (30 mg, 0.11 mmol), cesium carbonate (1.22 g, 3.47 mmol), potassium isopropenyl trifluoroborate (214 mg, 1.45 mmol) and THF/water 10:1 (4.5 mL) were treated as described in **GP4** (reaction time 5 h, reaction temperature 70 °C). The obtained yellowish oil was purified by column chromatography (silica gel, hexane/ethyl acetate = 90:10) to give **4** (267 mg, 89%) as a colourless oil. ^{1}H NMR ($CDCl_3$, 500 MHz): δ = 2.05 [dd, *J* = 1.0, 1.6 Hz, 3 H, =C(Ar)*Me*], 2.08 (s, 3 H, 5-H), 2.39 (dd, *J* = 4.0, 17.9 Hz, 1 H, 3-H), 2.76 (dd, *J* = 9.5, 13.7 Hz, 1 H, ArC*H*$_2$), 2.82 (dd, *J* = 9.7, 17.9 Hz, 1 H, 3-H), 3.08 (dd, *J* = 6.3, 13.7 Hz, 1 H, ArC*H*$_2$), 3.18 (dddd, *J* = 4.0, 6.3, 9.5, 9.7 Hz, 1 H, 2-H), 3.63 (s, 3 H, CO_2Me), 4.86 (qd, *J* = 1.0, 2.1 Hz, 1 H, =CH_2), 5.23 (qd, *J* = 1.6, 2.1 Hz, 1 H, =CH_2), 7.09-7.15, 7.17-7.20 (2 m, 2 × 2 H, Ar) ppm. ^{13}C NMR ($CDCl_3$, 125 MHz): δ = 25.2 [q, =C(Ar)*Me*], 30.0 (q, C-5), 34.7 (t, Ar*C*H$_2$), 41.4 (t, C-3), 44.3 (d, C-2), 115.7 (t, =CH_2), 126.6, 127.1, 128.6, 129.6, 134.8, 144.3, 145.2 [4 d, 3 s, *ArC*(Me)=CH$_2$], 51.9, 175.5 (q, s, CO_2Me), 206.8 (s, C-4) ppm. IR (neat): ṽ = 3075-2850 (=C-H, C-H), 1730, 1715 (C=O), 1640 (C=C) cm^{-1}. $C_{16}H_{20}O_3$ (260.3): calcd C 73.82, H 7.74; found C 73.71, H 7.37.

Methyl (2*RS*)-2-[(1*SR*)-2-oxocycloheptyl]-3-(2-vinylphenyl)propanoate (5a) and **methyl (2*RS*)-2-[(1*RS*)-2-oxocycloheptyl]-3-(2-vinylphenyl)propanoate (5b): SP9a/b** (1.20 g, 3.00 mmol), palladium(II) acetate (34 mg, 0.15 mmol), triphenylphosphine (79 mg, 0.30 mmol), cesium carbonate (3.18 g, 9.00 mmol), potassium vinyl trifluoroborate (442 mg, 3.30 mmol) and THF/water 10:1 (12 mL) were treated as described in **GP4** (reaction time 5 h, reaction temperature 70 °C). The obtained yellowish oil was purified by column chromatography (silica gel, hexane/ethyl acetate = 90:10), then HPLC (hexane/ethyl acetate = 85:15) to give **5a** (376 mg, 42%) and **5b** (350 mg, 39%) as colourless oils. **5a**: ^{1}H NMR ($CDCl_3$, 500 MHz): δ = 1.24-1.31, 1.33-1.43, 1.59-1.68, 1.73-1.78, 1.86-1.93 (5 m, 1 H, 2 H, 2 × 1 H, 3 H, 4'-H, 5'-H, 6'-H, 7'-H), 2.51-2.53, 2.80-2.84 (2 m, 2 × 1 H, 3'-H), 2.83 (dd, *J* = 9.6, 13.7 Hz, 1 H, 3-H), 2.91 (dd, *J* = 5.3, 13.7 Hz, 1 H, 3-H), 3.15 (ddd, *J* = 5.3, 8.9, 9.6 Hz, 1 H, 2-H), 3.49 (s, 3 H, CO_2Me), 5.35 (dd, *J* = 1.3, 11.0 Hz, 1 H, =CH_2), 5.67 (dd, *J* = 1.3, 17.1 Hz, 1 H, =CH_2), 7.05 (dd, *J* = 11.0, 17.1 Hz, 1 H, =C*H*Ar), 7.05-7.07, 7.13-7.21, 7.48-7.50 (3 m, 1 H, 2 H, 1 H, Ar) ppm. ^{13}C NMR ($CDCl_3$, 125 MHz): δ = 23.8, 28.9, 29.10, 29.12, (4 t, C-4', C-5', C-6', C-7'), 34.1 (t, C-3), 43.9 (t, C-3'), 48.8 (d, C-2), 53.5 (d, C-1'), 116.0 (t, =CH_2), 125.9, 127.1, 127.7, 130.1, 134.3, 136.2, 137.0 (5 d, 2 s, *Ar*CH=), 51.5, 174.7 (q, s, CO_2Me), 214.1 (s, C-2') ppm. IR (neat): $\tilde{\nu}$ = 3060-2855 (=C-H, C-H), 1730, 1700 (C=O), 1625, 1600, 1575 (C=C) cm^{-1}. $C_{19}H_{24}O_3$ (300.4): calcd C 75.97, H 8.05; found C 75.83, H 8.00.

5b: ^{1}H NMR ($CDCl_3$, 700 MHz): δ = 1.28-1.33, 1.42-1.48, 1.63-1.77, 1.81-1.93, 1.97-2.00 (5 m, 1 H, 2 H, 1 H, 3 H, 1 H, 4'-H, 5'-H, 6'-H, 7'-H), 2.43 (ddd, *J* = 4.0, 11.7, 15.6 Hz, 1 H, 3'-H), 2.56 (ddd, *J* = 4.2, 4.6, 15.6 Hz, 1 H, 3'-H), 2.86 (dd, *J* = 9.7, 13.6 Hz, 1 H, 3-H), 2.90-2.95 (m, 2 H, 2-H, 1'-H), 3.05 (dd, *J* = 4.4, 13.6 Hz, 1 H, 3-H), 3.44 (s, 3 H, CO_2Me), 5.32 (dd, *J* = 1.4, 10.9 Hz, 1 H, =CH_2), 5.65 (dd, *J* = 1.4, 17.3 Hz, 1 H, =CH_2), 6.99 (dd, *J* = 10.9, 17.3 Hz, 1 H, =C*H*Ar), 7.09-7.11, 7.16-7.21, 7.47-7.48 (3 m, 1 H, 2 H, 1 H, Ar) ppm. ^{13}C NMR ($CDCl_3$, 100 MHz): δ = 23.9, 28.9, 29.2, 29.4 (4 t, C-4', C-5', C-6', C-7'), 33.3 (t, C-3), 43.6 (t, C-3'), 48.6 (d, C-2), 53.5 (d, C-1'), 116.1 (t, =CH_2), 126.0, 127.0, 127.7, 130.3, 134.4, 136.3, 137.0 (5 d, 2 s, *Ar*CH=), 51.5, 175.3 (q, s, CO_2Me), 214.7 (s, C-2') ppm. IR (neat): $\tilde{\nu}$ = 3085-2855 (=C-H, C-H), 1730, 1700 (C=O), 1625, 1600, 1570 (C=C) cm^{-1}. HRMS (ESI) calcd for $C_{19}H_{24}O_3$: $[M+Na]^+$ = 323.1618; found: 323.1595.

Methyl (2*RS*)-3-(2-isopropenylphenyl)-2-[(1*SR*)-2-oxocyclohexyl]propanoate (7a) and **methyl (2*RS*)-3-(2-isopropenylphenyl)-2-[(1*RS*)-2-oxocyclohexyl]propanoate (7b): SP8a/b** (1.16 g, 3.00 mmol), palladium(II) acetate (34 mg, 0.15 mmol), triphenylphosphine (79 mg, 0.30 mmol), cesium carbonate (3.18 g, 9.00 mmol), potassium isopropenyl trifluoroborate (488 mg, 3.30 mmol) and THF/water 10:1 (12 mL) were treated as described in **GP4** (reaction time 5 h, reaction temperature 70 °C). The obtained yellowish oil was purified by column chromatography (silica gel, hexane/ethyl acetate = 90:10)), then HPLC (hexane/ethyl acetate = 87:13) to give **7a** (413 mg, 46%) and **7b** (170 mg, 19%) as colourless oils. **7a**: ^{1}H NMR ($CDCl_3$, 500 MHz): δ = 1.58-1.74, 1.84-1.89, 2.00-2.05 (3 m, 3 H, 1 H, 2 H, 4'-H, 5'-H, 6'-H), 2.08 [dd, *J* = 1.0, 1.6 Hz, 3 H, =C(Ar)*Me*], 2.26-2.33, 2.40-2.45 (2 m, 2 × 1 H, 3'-H), 2.49-2.54 (m, 1 H, 1'-H), 2.89 (dd, *J* = 9.3, 13.9 Hz, 1 H, 3-H), 2.99 (dd, *J* = 6.3, 13.9 Hz, 1 H, 3-H), 3.32 (ddd, *J* = 6.3, 7.4, 9.3 Hz, 1 H, 2-H), 3.51 (s, 3 H, CO_2Me), 4.86 (qd, *J* = 1.0, 2.1 Hz, 1 H, =CH_2), 5.23 (qd, *J* = 1.6, 2.1 Hz, 1 H, =CH_2), 7.07-7.10, 7.13-7.21 (2 m, 1 H, 3 H, Ar) ppm. ^{13}C NMR ($CDCl_3$, 125 MHz): δ = 24.8 (t, C-4'), 25.1 [q, =C(Ar)*Me*], 27.7, 30.9 (2 t, C-5', C-6'), 33.6 (t, C-3), 42.2 (t, C-3'), 46.0 (d, C-2), 52.4 (d, C-1'), 115.4 (t, =CH_2), 126.3, 126.9, 128.4, 129.5, 135.5, 144.2, 145.4 [4 d, 3 s, *ArC*(Me)=CH_2], 51.4, 174.7 (q, s, CO_2Me), 211.0 (s, C-2') ppm. IR (neat): $\tilde{v}$ = 3075-2865 (=C-H, C-H), 1735, 1710 (C=O), 1640 (C=C) cm^{-1}. MS (EI = 70 eV): *m/z* (%) = 300 (22) $[M]^+$, 269 (7), 203 (41), 143 (100), 98 (81), 43 (54). HRMS (ESI) calcd for $C_{19}H_{24}O_3$: $[M+Na]^+$ = 323.1618; found: 323.1624. $C_{19}H_{24}O_3$ (300.4): calcd C 75.97, H 8.05; found C 76.29, H 8.00.

7b: ^{1}H NMR ($CDCl_3$, 500 MHz): δ = 1.42-1.50, 1.62-1.73, 1.90-1.95, 2.06-2.11 (4 m, 1 H, 2 H, 2 × 1 H, 4'-H, 5'-H, 6'-H), 2.05 [dd, *J* = 1.0, 1.6 Hz, 3 H, =C(Ar)*Me*], 2.23-2.42 (m, 3 H, 3'-H, 6'-H), 2.69 (dd, *J* = 10.6, 13.5 Hz, 1 H, 3-H), 2.76 (dddd, *J* = 1.1, 5.5, 8.4, 12.7 Hz, 1 H, 1'-H), 2.85 (ddd, *J* = 5.0, 8.4, 10.6 Hz, 1 H, 2-H), 3.05 (dd, *J* = 5.0, 13.5 Hz, 1 H, 3-H), 3.42 (s, 3 H, CO_2Me), 4.86 (qd, *J* = 1.0, 2.1 Hz, 1 H, =CH_2), 5.19 (qd, *J* = 1.6, 2.1 Hz, 1 H, =CH_2), 7.08-7.18 (m, 4 H, Ar) ppm. ^{13}C NMR ($CDCl_3$, 125 MHz): δ = 25.29, 25.33, (2 t, C-4', C-5'), 27.7 [q, =C(Ar)*Me*], 31.3 (t, C-6'), 33.3 (t, C-3), 42.2 (t, C-3'), 46.5 (d, C-2), 53.2 (d, C-1'), 115.3 (t, =CH_2), 126.4, 126.7, 128.3, 129.8, 135.3, 144.0, 145.4 [4 d, 3 s, *ArC*(Me)=CH_2], 51.3, 175.9 (q, s, CO_2Me), 211.2 (s, C-2') ppm.

IR (neat): $\tilde{v}$ = 3060-2860 (=C-H, C-H), 1730, 1710 (C=O), 1640 (C=C) cm^{-1}. MS (EI = 70 eV): *m/z* (%) = 300 (7) $[M]^+$, 203 (29), 171 (21), 143 (100), 128 (45), 115 (38). HRMS (80 eV) calcd for $C_{19}H_{24}O_3$: $[M]^+$ = 300.1726; found: 300.1708.

Methyl 2-(2-isopropenylbenzyl)-5-methyl-4-oxohexanoate (11): Methyl 2-(2-iodobenzyl)-5-methyl-4-oxohexanoate (0.75 g, 2.00 mmol), palladium(II) acetate (23 mg, 0.10 mmol), triphenylphosphine (53 mg, 0.20 mmol), cesium carbonate (1.96 g, 6.00 mmol), potassium isopropenyl trifluoroborate (326 mg, 2.20 mmol) and THF/water 10:1 (8 mL) were treated as described in **GP4** (reaction time 12 h, reaction temperature 70 °C). The obtained yellowish oil was purified by column chromatography (silica gel, hexane/ethyl acetate = 90:10) to give **11** (434 mg, 75%) as a colourless oil. ^{1}H NMR ($CDCl_3$, 500 MHz): δ = 1.01, 1.05 (2 d, *J* = 6.9 Hz, 2 × 3 H, 5-Me, 6-H), 2.04 [dd, *J* = 1.0, 1.6 Hz, 3 H, =C(Ar)*Me*], 2.39 (dd, *J* = 4.0, 17.8 Hz, 1 H, 3-H), 2.52 (sept, *J* = 6.9 Hz, 1 H, 5-H), 2.76 (dd, *J* = 9.4, 13.8 Hz, 1 H, ArCH_2), 2.85 (dd, *J* = 9.6, 17.8 Hz, 1 H, 3-H), 3.06 (dd, *J* = 6.5, 13.8 Hz, 1 H, ArCH_2), 3.19 (dddd, *J* = 4.0, 6.5, 9.4, 9.6 Hz, 1 H, 2-H), 3.62 (s, 3 H, CO_2Me), 4.86 (qd, *J* = 1.0, 2.0 Hz, 1 H, =CH_2), 5.23 (qd, *J* = 1.6, 2.0 Hz, 1 H, =CH_2), 7.10-7.19 (m, 4 H, Ar) ppm. ^{13}C NMR ($CDCl_3$, 125 MHz): δ = 18.1, 18.2 (2 q, 5-Me, C-6), 25.3 [q, =C(Ar)*Me*], 34.9 (t, Ar*C*H_2), 40.9 (d, C-5), 41.3 (t, C-3), 41.4 (d, C-2), 115.7 (t, =CH_2), 126.6, 127.1, 128.6, 129.6, 135.0, 144.3, 145.3 [4 d, 3 s, *ArC*(Me)=CH_2], 51.9, 175.6 (q, s, CO_2Me), 212.8 (s, C-4) ppm. IR (neat): $\tilde{v}$ = 3075-2875 (=C-H, C-H), 1735, 1715 (C=O), 1640 (C=C) cm^{-1}. MS (EI = 70 eV): *m/z* (%) = 288 (22) $[M]^+$, 257 (10), 213 (16), 203 (17), 143 (100), 91 (21), 71 (36), 43 (84). $C_{18}H_{24}O_3$ (288.4): calcd C 74.97, H 8.39; found C 74.60, H 7.85.

Methyl 2-(2-isopropenylbenzyl)-3,3,5-trimethyl-4-oxohexanoate (14): SP11 (0.50 g, 1.24 mmol), palladium(II) acetate (14 mg, 0.062 mmol), triphenylphosphine (33 mg, 0.13 mmol), cesium carbonate (1.32 g, 3.73 mmol), potassium isopropenyl trifluoroborate (230 mg, 1.55 mmol) and THF/water 10:1 (5.5 mL) were treated as described in **GP4** (reaction time 5 h, reaction temperature 70 °C). The obtained yellowish oil was purified by column chromatography (silica gel, hexane/ethyl acetate = 90:10) to give **14** (303 mg, 77%) as a colourless oil. ^{1}H NMR ($CDCl_3$, 500 MHz): δ = 1.06, 1.07 (2 d, *J* = 7.0

Hz, 2 × 3 H, 5-Me, 6-H), 1.25, 1.31 (2 s, 2 × 3 H, 3-Me), 2.05 [s, 3 H, =C(Ar)*Me*], 2.75 (dd, *J* = 3.3, 13.7 Hz, 1 H, ArCH_2), 2.89 (dd, *J* = 11.8, 13.7 Hz, 1 H, ArCH_2), 3.14 (sept, *J* = 6.7 Hz, 1 H, 5-H), 3.19 (dd, *J* = 3.3, 11.8 Hz, 1 H, 2-H), 3.44 (s, 3 H, CO_2Me), 4.84, 5.21 (2 s, 2 × 1 H, =CH_2), 7.05-7.08, 7.13-7.19 (2 m, 1 H, 3 H, Ar) ppm. ^{13}C NMR ($CDCl_3$, 125 MHz): δ = 20.1, 20.4 (2 q, 5-Me, C-6), 21.3, 22.6 (2 q, 3-Me), 25.3 [q, =C(Ar)*Me*], 31.3 (t, Ar*C*H_2), 34.7 (d, C-5), 50.5 (s, C-3), 52.2 (d, C-2), 115.5 (t, =CH_2), 126.3, 126.9, 128.3, 129.5, 134.7, 144.1, 145.3 [4 d, 3 s, *ArC*(Me)=CH_2], 51.1, 174.5 (q, s, CO_2Me), 218.1 (s, C-4) ppm. IR (neat): ṽ = 3060-2875 (=C-H, C-H), 1730, 1700 (C=O), 1600 (weak, C=C) cm^{-1}. MS (EI = 70 eV): *m/z* (%) = 316 (1) $[M]^+$, 301 (6) $[M - CH_3]^+$, 273 (15), 157 (15), 119 (18), 114 (33), 91 (22), 71 (45), 43 (100). HRMS (80 eV) calcd for $C_{19}H_{25}O_3$: $[M - CH_3]^+$ = 301.1804; found: 301.1801.

Methyl 5-methyl-4-oxo-2-[2-(1-phenylethenyl)benzyl]hexanoate (16): Methyl 2-(2-iodobenzyl)-5-methyl-4-oxohexanoate (400 mg, 1.07 mmol), palladium(II) acetate (12 mg, 0.053 mmol), triphenylphosphine (56 mg, 0.21 mmol), potassium carbonate (0.22 g, 1.60 mmol), 1-phenylvinylboronic acid (174 mg, 1.18 mmol) and DMF (6 mL) were treated as described in **GP4** (reaction time 72 h, reaction temperature 70 °C). The obtained yellowish oil was purified by column chromatography (silica gel, hexane/ethyl acetate = 90:10) to give **14** (325 mg, 87%) as a colourless oil. ^{1}H NMR ($CDCl_3$, 500 MHz): δ = 0.98, 1.01 (2 d, *J* = 7.0 Hz, 2 × 3 H, 5-Me, 6-H), 2.23 (dd, *J* = 3.8, 17.8 Hz, 1 H, 3-H), 2.45 (sept, *J* = 7.0 Hz, 1 H, 5-H), 2.48 (dd, *J* = 9.0, 14.0 Hz, 1 H, ArCH_2), 2.67 (dd, *J* = 10.1, 17.8 Hz, 1 H, 3-H), 2.76 (dd, *J* = 6.5, 14.0 Hz, 1 H, ArCH_2), 3.06 (dddd, *J* = 3.8, 6.5, 9.0, 10.1 Hz, 1 H, 2-H), 3.55 (s, 3 H, CO_2Me), 5.24 (d, *J* = 1.3 Hz, 1 H, =CH_2), 5.81 (d, *J* = 1.3 Hz, 1 H, =CH_2), 7.23-7.34 (m, 9 H, Ar) ppm. ^{13}C NMR ($CDCl_3$, 125 MHz): δ = 18.1, 18.2 (2 q, 5-Me, C-6), 35.4 (t, Ar*C*H_2), 40.8, 41.2 (2 d, C-2, C-5), 41.3 (t, C-3), 116.0 (t, =CH_2), 126.6, 126.8, 127.8, 127.9, 128.5, 129.9, 130.9, 136.7, 140.6, 141.9, 149.0 [7 d, 4 s, *ArC*(=CH_2)*Ph*], 51.7, 175.5 (q, s, CO_2Me), 212.7 (s, C-4) ppm. IR (neat): ṽ = 3080-2875 (=C-H, C-H), 1735, 1715 (C=O), 1615 (C=C) cm^{-1}. MS (EI = 70 eV): *m/z* (%) = 350 (6) $[M]^+$, 289 (31), 247 (58), 205 (40), 192 (75) 116 (31), 115 (31), 71 (45), 43 (100). HRMS (80 eV) calcd for $C_{23}H_{26}O_3$: $[M]^+$ = 350.1882; found: 350.1870.

Methyl 4-oxo-2-{2-[(1*E*)-propen-1-yl]benzyl}pentanoate (18): Methyl 2-(2-iodobenzyl)-4-oxopentanoate (400 mg, 1.16 mmol), palladium(II) acetate (13 mg, 0.058 mmol), triphenylphosphine (61 mg, 0.23 mmol), potassium carbonate (0.24 g, 1.73 mmol), (*E*)-propen-1-ylboronic acid (109 mg, 1.27 mmol) and DMF (4 mL) were treated as described in **GP4** (reaction time 72 h, reaction temperature 70 °C). The obtained yellowish oil was purified by column chromatography (silica gel, hexane/ethyl acetate = 90:10) to give **18** (279 mg, 93%) as a colourless oil. ^{1}H NMR ($CDCl_3$, 500 MHz): δ = 1.91 (dd, *J* = 1.8, 6.6 Hz, 3 H, =CH*Me*), 2.07 (s, 3 H, 5-H), 2.45 (dd, *J* = 4.1, 18.1 Hz, 1 H, 3-H), 2.74 (dd, *J* = 11.0, 15.7 Hz, 1 H, ArCH_2), 2.83 (dd, *J* = 8.6, 18.1 Hz, 1 H, 3-H), 3.10 (dd, *J* = 6.1, 15.7 Hz, 1 H, ArCH_2), 3.11 (dddd, *J* = 4.1, 6.1, 8.6, 11.0 Hz, 1 H, 2-H), 3.64 (s, 3 H, CO_2Me), 6.13 (qd, *J* = 6.6, 15.5 Hz, 1 H, =C*H*Me), 6.64 (qd, *J* = 1.8, 15.5 Hz, 1 H, =C*H*Ar), 7.04-7.05, 7.11-7.20, 7.41-7.43 (3 m, 1 H, 2 H, 1 H, Ar) ppm. ^{13}C NMR ($CDCl_3$, 125 MHz): δ = 18.9 (q, =CH*Me*), 30.0 (q, C-5), 34.9 (t, Ar*C*H_2), 41.3 (t, C-3), 44.0 (d, C-2), 126.2, 126.9, 127.1, 128.2, 128.2, 130.3, 135.2, 137.3 (6 d, 2 s, ArCH=CH), 51.9, 175.4 (q, s, CO_2Me), 206.7 (s, C-4) ppm. IR (neat): $\tilde{\nu}$ = 3095-2855 (=C-H, C-H), 1735, 1720 (C=O), 1650 (C=C) cm^{-1}. $C_{16}H_{20}O_3$ (260.3): calcd C 73.82, H 7.74; found C 73.27, H 7.57.

Methyl 4-oxo-2-{2-[(1*E*)-propen-1-yl]benzyl}hexanoate (21): SP7 (0.54 g, 1.50 mmol), Palladium(II) acetate (17 mg, 0.076 mmol), triphenylphosphine (79 mg, 0.30 mmol), potassium carbonate (0.31 g, 2.25 mmol), (*E*)-propen-1-ylboronic acid (142 mg, 1.65 mmol) and DMF (4 mL) were treated as described in **GP4** (reaction time 12 h, reaction temperature 70 °C). The obtained yellowish oil was purified by column chromatography (silica gel, hexane/ethyl acetate = 90:10) to give **21** (343 mg, 83%) as a colourless oil. ^{1}H NMR ($CDCl_3$, 500 MHz): δ = 0.99 (t, *J* = 7.3 Hz, 3 H, 6-H), 1.91 (dd, *J* = 1.7, 6.6 Hz, 3 H, =CH*Me*), AB part of ABX_3-system (δ_A = 2.35, δ_B = 2.40, J_{AB} = 17.6 Hz, J_{AX} = J_{BX} = 7.3 Hz, 2 H, 5-H), 2.42 (dd, *J* = 4.4, 17.8 Hz, 1 H, 3-H), 2.75 (dd, *J* = 8.5, 13.1 Hz, 1 H, ArCH_2), 2.80 (dd, *J* = 9.0, 17.8 Hz, 1 H, 3-H), 3.10 (dd, *J* = 6.0, 13.1 Hz, 1 H, ArCH_2), 3.10-3.16 (m, 1 H, 2-H), 3.65 (s, 3 H, CO_2Me), 6.12 (qd, *J* = 6.6, 15.4 Hz, 1 H, =C*H*Me), 6.64 (qd, *J* = 1.7, 15.4 Hz, 1 H, =C*H*Ar), 7.04-7.06, 7.11-7.19, 7.41-7.43 (3

m, 1 H, 2 H, 1 H, Ar) ppm. ^{13}C NMR ($CDCl_3$, 125 MHz): δ = 7.7 (q, C-6), 19.0 (q, =CH*Me*), 35.1 (t, Ar*C*H_2), 36.0 (t, C-5), 41.3 (d, C-2), 42.8 (t, C-3), 126.3, 126.9, 127.1, 128.2, 128.3, 130.3, 135.3, 137.3 (6 d, 2 s, ArCH=CH), 52.0, 175.5 (q, s, CO_2Me), 209.5 (s, C-4) ppm. IR (neat): ṽ = 3060-2840 (=C-H, C-H), 1730, 1710 (C=O), 1600 (br, C=C) cm^{-1}. MS (EI = 70 eV): *m/z* (%) = 274 (7) $[M]^+$, 259 (10), 187 (28), 143 (50), 129 (67) 115 (44), 91 (29), 57 (100). HRMS (80 eV) calcd for $C_{17}H_{22}O_3$: $[M]^+$ = 274.15689; found: 274.1566.

Methyl 5-methyl-4-oxo-2-{2-[(1*E*)-propen-1-yl]benzyl}hexanoate (24): Methyl 2-(2-iodobenzyl)-5-methyl-4-oxohexanoate (0.70 g, 1.87 mmol), palladium(II) acetate (39 mg, 0.17 mmol), triphenylphosphine (98 mg, 0.37 mmol), potassium carbonate (0.39 g, 2.80 mmol), (*E*)-propen-1-ylboronic acid (177 mg, 2.06 mmol) and DMF (8 mL) were treated as described in **GP4** (reaction time 72 h, reaction temperature 70 °C). The obtained yellowish oil was purified by column chromatography (silica gel, hexane/ethyl acetate = 90:10) to give **24** (474 mg, 88%) as a colourless oil. ^{1}H NMR ($CDCl_3$, 500 MHz): δ = 1.02, 1.06 (2 d, *J* = 6.9 Hz, 2 × 3 H, 5-Me, 6-H), 1.91 (dd, *J* = 1.8, 6.6 Hz, 3 H, =CH*Me*), 2.46 (dd, *J* = 4.3, 17.9 Hz, 1 H, 3-H), 2.51 (sept, *J* = 6.9 Hz, 1 H, 5-H), 2.75 (dd, *J* = 8.4, 13.1 Hz, 1 H, ArC*H*$_2$), 2.87 (dd, *J* = 8.8, 17.9 Hz, 1 H, 3-H), 3.09 (dd, *J* = 6.2, 13.1 Hz, 1 H, ArC*H*$_2$), 3.13 (dddd, *J* = 4.3, 6.2, 8.4, 8.8 Hz, 1 H, 2-H), 3.63 (s, 3 H, CO_2Me), 6.12 (qd, *J* = 6.6, 15.5 Hz, 1 H, =C*H*Me), 6.65 (qd, *J* = 1.8, 15.5 Hz, 1 H, =C*H*Ar), 7.04-7.06, 7.11-7.19, 7.40-7.42 (3 m, 1 H, 2 H, 1 H, Ar) ppm. ^{13}C NMR ($CDCl_3$, 125 MHz): δ = 18.1, 18.3 (2 q, 5-Me, C-6), 18.9 (q, =CH*Me*), 35.1 (t, Ar*C*H_2), 40.9 (d, C-5), 41.0 (t, C-3), 41.3 (d, C-2), 126.2, 126.9, 127.1, 128.1, 128.3, 130.2, 135.3, 137.3 (6 d, 2 s, ArCH=CH), 51.9, 175.5 (q, s, CO_2Me), 212.7 (s, C-4) ppm. IR (neat): ṽ = 3090-2850 (=C-H, C-H), 1735, 1710 (C=O), 1655 (C=C) cm^{-1}. MS (EI = 70 eV): *m/z* (%) = 288 (26) $[M]^+$, 257 (8), 203 (17), 157 (13) 143 (100), 91 (33), 71 (30), 43 (87). $C_{18}H_{24}O_3$ (288.4): calcd C 74.97, H 8.39; found C 75.43, H 8.05.

Methyl (2*RS*)-2-[(1*SR*)-2-oxocyclohexyl]-3-{2-[(1*E*)-propen-1-yl]phenyl}propanoate (26a) and **methyl (2*RS*)-2-[(1*RS*)-2-oxocyclohexyl]-3-{2-[(1*E*)-propen-1-yl]phenyl}propanoate (26b): SP8a/b** (0.80 g, 2.07 mmol), palladium(II) acetate (23

mg, 0.10 mmol), triphenylphosphine (109 mg, 0.42 mmol), potassium carbonate (0.43 g, 3.10 mmol), (*E*)-propen-1-ylboronic acid (196 mg, 2.28 mmol) and DMF (12 mL) were treated as described in **GP4** (reaction time 12 h, reaction temperature 70 °C). The obtained yellowish oil was purified by column chromatography (silica gel, hexane/ethyl acetate = 90:10) to give **26a** (124 mg, 20%) and **26b** (404 mg, 65%) as colourless oils.
26a: ^{1}H NMR ($CDCl_3$, 500 MHz): δ = 1.54-1.75, 1.87-1.90 (2 m, 3 H, 1 H, 4'-H, 5'-H, 6'-H), 1.96 (dd, *J* = 1.3, 6.6 Hz, 3 H, =CH*Me*), 2.03-2.10, 2.32-2.38, 2.43-2.47 (3 m, 2 H, 2 × 1 H, 3'-H, 5'-H, 6'-H), 2.60 (ddd, *J* = 5.4, 8.1, 11.5 Hz, 1 H, 1'-H), 2.79 (dd, *J* = 9.7, 13.4 Hz, 1 H, 3-H), 3.08 (dd, *J* = 5.2, 13.4 Hz, 1 H, 3-H), 3.15 (ddd, *J* = 5.2, 8.1, 9.7 Hz, 1 H, 2-H), 3.49 (s, 3 H, CO_2Me), 6.15 (qd, *J* = 6.6, 15.3 Hz, 1 H, =C*H*Me), 6.89 (qd, *J* = 1.3, 15.3 Hz, 1 H, =C*H*Ar), 7.05-7.16, 7.42-7.43 (2 m, 3 H, 1 H, Ar) ppm. ^{13}C NMR ($CDCl_3$, 125 MHz): δ = 19.0 (q, Me), 25.2 (t, C-4'), 28.2, 31.9 (2 t, C-5', C-6'), 34.4 (t, C-3), 42.6 (t, C-3'), 46.6 (d, C-2), 52.6 (d, C-1'), 125.8, 126.7, 126.9, 127.5, 128.6, 130.0, 135.9, 137.2 (6 d, 2 s, ArCH=CH), 51.4, 174.8 (q, s, CO_2Me), 211.3 (s, C-2') ppm. IR (neat): $\tilde{\nu}$ = 3065-2875 (=C-H, C-H), 1730, 1715 (C=O), 1600 (C=C) cm^{-1}. HRMS (ESI) calcd for $C_{19}H_{24}O_3$: $[M+Na]^+$ = 323.1618; found: 323.1627.
26b: ^{1}H NMR ($CDCl_3$, 500 MHz): δ = 1.53-1.73 (m, 3 H, 4'-H, 5'-H, 6'-H), 1.90 (dd, *J* = 1.5, 6.6 Hz, 3 H, =CH*Me*), 1.94-1.98, 2.07-2.12, 2.29-2.45 (3 m, 2 × 1 H, 3 H, 3'-H, 4'-H, 5'-H, 6'-H), 2.74 (dd, *J* = 10.4, 13.3 Hz, 1 H, 3-H), 2.74-2.79 (m, 1 H, 1'-H), 2.88 (ddd, *J* = 4.8, 7.2, 10.4 Hz, 1 H, 2-H), 3.01 (dd, *J* = 4.8, 13.3 Hz, 1 H, 3-H), 3.43 (s, 3 H, CO_2Me), 6.11 (qd, *J* = 6.6, 15.5 Hz, 1 H, =C*H*Me), 6.67 (qd, *J* = 1.5, 15.5 Hz, 1 H, =C*H*Ar), 7.04-7.17, 7.39-7.41 (2 m, 3 H, 1 H, Ar) ppm. ^{13}C NMR ($CDCl_3$, 125 MHz): δ = 18.9 (q, Me), 25.3, 27.7 (2 t, C-4', C-5'), 31.0 (t, C-6'), 32.9 (t, C-3), 42.2 (t, C-3'), 46.1 (d, C-2), 52.8 (d, C-1'), 126.0, 126.7, 126.9, 127.7, 128.5, 130.2, 135.8, 137.1 (6 d, 2 s, ArCH=CH], 51.4, 175.6 (q, s, CO_2Me), 211.0 (s, C-2') ppm. IR (neat): $\tilde{\nu}$ = 3060-2845 (=C-H, C-H), 1730, 1710 (C=O), 1600 (C=C) cm^{-1}. MS (EI = 70 eV): *m/z* (%) = 300 (6) $[M]^+$, 203 (31), 171 (30), 143 (100), 98 (40), 91 (25). HRMS (80 eV) calcd for $C_{19}H_{24}O_3$: $[M]^+$ = 300.17255; found: 300.17224.

Methyl 5-methyl-4-oxo-2-{2-[(1*Z*)-propen-1-yl]benzyl}hexanoate (30): Methyl 2-(2-iodobenzyl)-5-methyl-4-oxohexanoate (0.56 g, 1.50 mmol), palladium(II) acetate (17

mg, 0.076 mmol), triphenylphosphine (79 mg, 0.30 mmol), potassium carbonate (0.31 g, 2.25 mmol), (*Z*)-propen-1-ylboronic acid (142 mg, 1.65 mmol) and DMF (6 mL) were treated as described in **GP4** (reaction time 72 h, reaction temperature 70 °C). The obtained yellowish oil was purified by column chromatography (silica gel, hexane/ethyl acetate = 90:10) to give **30** (403 mg, 93%) as a colourless oil. ^{1}H NMR ($CDCl_3$, 500 MHz): δ = 1.01, 1.05 (2 d, *J* = 6.9 Hz, 2 × 3 H, 5-Me, 6-H), 1.71 (dd, *J* = 1.8, 7.0 Hz, 3 H, =CH*Me*), 2.40 (dd, *J* = 4.1, 17.9 Hz, 1 H, 3-H), 2.50 (sept, *J* = 6.9 Hz, 1 H, 5-H), 2.71 (dd, *J* = 9.2, 13.6 Hz, 1 H, ArCH_2), 2.84 (dd, *J* = 9.4, 17.9 Hz, 1 H, 3-H), 3.00 (dd, *J* = 6.5, 13.6 Hz, 1 H, ArCH_2), 3.13 (dddd, *J* = 4.1, 6.5, 9.2, 9.4 Hz, 1 H, 2-H), 3.61 (s, 3 H, CO_2Me), 5.87 (qd, *J* = 7.0, 11.4 Hz, 1 H, =C*H*Me), 6.52 (qd, *J* = 1.8, 11.4 Hz, 1 H, =C*H*Ar), 7.11-7.13, 7.15-7.20 (2 m, 1 H, 3 H, Ar) ppm. ^{13}C NMR ($CDCl_3$, 125 MHz): δ = 14.4 (q, =CH*Me*), 18.1, 18.3 (2 q, 5-Me, C-6), 35.3 (t, Ar*C*H_2), 40.9 (d, C-5), 41.0 (d, C-2), 41.1 (t, C-3), 126.4, 127.0, 128.0, 128.5, 129.9, 130.0, 136.8, 136.9 (6 d, 2 s, ArCH=CH), 51.8, 175.6 (q, s, CO_2Me), 212.7 (s, C-4) ppm. IR (neat): ṽ = 3090-2875 (=C-H, C-H), 1735, 1710 (C=O), 1645 (br, C=C) cm^{-1}. $C_{18}H_{24}O_3$ (288.4): calcd C 74.97, H 8.39; found C 74.89, H 8.53.

Methyl 2-{2-[(*E*)-2-cyclopropylvinyl]benzyl}-5-methyl-4-oxohexanoate (33): Methyl 2-(2-iodobenzyl)-5-methyl-4-oxohexanoate (0.56 g, 1.50 mmol), (triphenylphosphine)-palladium(II) dichloride (32 mg, 0.046 mmol), 1,1′-bis(diphenylphosphino)ferrocene (25 mg, 0.045 mmol), potassium carbonate (0.62 g, 4.50 mmol), (*E*)-2-cyclopropyl-vinylboronic acid pinacol ester (320 mg, 1.65 mmol) and DMF (6 mL) were treated as described in **GP4** (reaction time 18 h, reaction temperature 80 °C). The obtained yellowish oil was purified by column chromatography (silica gel, hexane/ethyl acetate = 90:10) to give **33** (423 mg, 90%) as a colourless oil. ^{1}H NMR ($CDCl_3$, 500 MHz): δ = 0.51-0.54, 0.82-0.86 (2 m, 2 × 2 H, 2'-H, 3'-H), 1.03, 1.07 (2 d, *J* = 6.9 Hz, 2 × 3 H, 5-Me, 6-H), 1.62 (dtt, *J* = 4.3, 8.5, 8.8 Hz, 1 H, 1'-H), 2.48 (dd, *J* = 4.2, 17.9 Hz, 1 H, 3-H), 2.53 (sept, *J* = 6.9 Hz, 1 H, 5-H), 2.75-2.80 (m, 1 H, ArCH_2), 2.87 (dd, *J* = 8.8, 17.9 Hz, 1 H, 3-H), 3.09-3.17 (m, 2 H, 2-H, ArCH_2), 3.64 (s, 3 H, CO_2Me), 5.63 (dd, *J* = 8.8, 15.5 Hz, 1 H, 1'-C*H*=), 6.71 (d, *J* = 15.5 Hz, 1 H, =C*H*Ar), 7.04-7.17, 7.37-7.39 (2 m, 3 H, 1 H, Ar) ppm. ^{13}C NMR ($CDCl_3$, 125 MHz): δ = 7.5 (t, C-2', C-3'), 15.0 (d, C-1'), 18.1, 18.3

(2 q, 5-Me, C-6), 35.1 (t, Ar*C*H_2), 40.9 (d, C-5), 41.0 (t, C-3), 41.3 (d, C-2), 124.4 (d, =*C*HAr), 137.2 (d, 1'-*C*H=), 125.8, 126.7, 127.1, 130.3, 135.1, 137.0 (4 d, 2 s, Ar), 51.9, 175.5 (q, s, CO_2Me), 212.8 (s, C-4) ppm. IR (neat): ṽ = 3085-2850 (=C-H, C-H), 1735, 1710 (C=O), 1645 (C=C) cm^{-1}. $C_{20}H_{26}O_3$ (314.4): calcd C 76.40, H 8.33; found C 76.24, H 8.28.

Methyl 5-methyl-4-oxo-2-{2-[2-phenyl-(1*E*)-ethenyl]benzyl}hexanoate (36): Methyl 2-(2-iodobenzyl)-5-methyl-4-oxohexanoate (200 mg, 0.53 mmol), palladium(II) acetate (6 mg, 0.027 mmol), triphenylphosphine (28 mg, 0.11 mmol), potassium carbonate (0.11 g, 0.80 mmol), (*E*)-2-phenylvinylboronic acid (87 mg, 0.59 mmol) and DMF (3 mL) were treated as described in **GP4** (reaction time 12 h, reaction temperature 70 °C). The obtained yellowish oil was purified by column chromatography (silica gel, hexane/ethyl acetate = 90:10) to give **30** (187 mg, 100%) as a colourless oil. ^{1}H NMR ($CDCl_3$, 500 MHz): δ = 1.04, 1.07 (2 d, *J* = 7.0 Hz, 2 × 3 H, 5-Me, 6-H), 2.54 (sept, *J* = 7.0 Hz, 1 H, 5-H), 2.57 (dd, *J* = 4.9, 18.0 Hz, 1 H, 3-H), 2.89 (dd, *J* = 8.4, 18.0 Hz, 1 H, 3-H), 2.83-2.90, 3.13-3.21 (2 m, 1 H, 2 H, ArC*H_2*, 2-H), 3.62 (s, 3 H, CO_2Me), 7.02, 7.46 (2 d, *J* = 16.1 Hz, 2 × 1 H, =CH), 7.11-7.28, 7.35-7.39, 7.57-7.65 (3 m, 4 H, 2 H, 3 H, Ar) ppm. ^{13}C NMR ($CDCl_3$, 125 MHz): δ = 18.1, 18.3 (2 q, 5-Me, C-6), 35.3 (t, Ar*C*H_2), 40.9, 41.7 (2 d, C-2, C-5), 41.1 (t, C-3), 125.8, 126.0, 126.8, 127.3, 127.6, 127.8, 128.8, 130.5, 130.9, 136.5, 136.6, 137.6 (9 d, 3 s, ArCH=CHPh), 52.0, 175.3 (q, s, CO_2Me), 212.5 (s, C-4) ppm. IR (neat): ṽ = 3060-2875 (=C-H, C-H), 1730, 1710 (C=O), 1600 (C=C) cm^{-1}. MS (EI = 70 eV): *m/z* (%) = 350 (27) $[M]^+$, 264 (11), 205 (100), 192 (32), 115 (57), 91 (50), 43 (98). HRMS (80 eV) calcd for $C_{23}H_{26}O_3$: $[M]^+$ = 350.18820; found: 350.18766.

Methyl (2*RS*)-2-[(1*SR*)-2-oxocyclohexyl]-3-{2-[(*E*)-2-phenylvinyl]phenyl}propanoate (38a) and **methyl (2*RS*)-2-[(1*RS*)-2-oxocyclohexyl]-3-{2-[(*E*)-2-phenylvinyl]phenyl}propanoate (38b): SP8a/b** (206 mg, 0.53 mmol), palladium(II) acetate (6 mg, 0.027 mmol), triphenylphosphine (28 mg, 0.11 mmol), potassium carbonate (0.11 g, 0.80 mmol), (*E*)-2-phenylvinylboronic acid (87 mg, 0.59 mmol) and DMF (3 mL) were treated as described in **GP4** (reaction time 72 h, reaction temperature 70 °C). The obtained yellowish oil was purified by column chromatography (silica gel,

hexane/ethyl acetate = 90:10) to give **38a** (90 mg, 47%) and **38b** (92 mg, 48%) as colourless crystals. (**38a**: mp 117-119 °C, **38b**: mp 104-106 °C). **38a**: ^{1}H NMR ($CDCl_3$, 500 MHz): δ = 1.48-1.57, 1.61-1.74, 1.82-1.87, 1.99-2.04, 2.05-2.11, 2.32-2.39, 2.44-2.48 (7 m, 1 H, 2 H, 5 × 1 H, 3'-H, 4'-H, 5'-H, 6'-H), 2.66 (ddd, *J* = 5.1, 8.8, 12.3 Hz, 1 H, 1'-H), 2.84 (dd, *J* = 10.3, 13.4 Hz, 1 H, 3-H), 3.16 (ddd, *J* = 4.7, 8.8, 10.3 Hz, 1 H, 2-H), 3.28 (dd, *J* = 4.7, 13.4 Hz, 1 H, 3-H), 3.46 (s, 3 H, CO_2Me), 7.06 (d, *J* = 16.1 Hz, 1 H, =CH), 7.11-7.28, 7.37-7.41, 7.65-7.72 (3 m, 4 H, 2 H, 3 H, Ar), 7.77 (d, *J* = 16.1 Hz, 1 H, =CH) ppm. ^{13}C NMR ($CDCl_3$, 125 MHz): δ = 25.3 (t, C-4'), 28.4 (t, C-5'), 32.4 (t, C-6'), 34.9 (t, C-3), 42.8 (t, C-3'), 47.4 (d, C-1'), 52.9 (d, C-2), 125.6, 126.1, 127.0, 127.1, 127.5, 127.6, 128.8, 130.4, 130.5 (9 d, ArCH=CHPh), 136.5, 137.1, 137.8 (3 s, Ar), 51.4, 174.8 (q, s, CO_2Me), 211.1 (s, C-2') ppm. IR (KBr): $\tilde{\nu}$ = 3060-2860 (=C-H, C-H), 1735, 1710 (C=O), 1600 (C=C) cm^{-1}. MS (EI = 70 eV): *m/z* (%) = 362 (40) $[M]^+$, 264 (15), 205 (100), 178 (23), 159 (18), 115 (45), 91 (71). $C_{24}H_{26}O_3$ (362.5): calcd C 79.53, H 7.23; found C 78.60, H 7.22.

38b: ^{1}H NMR ($CDCl_3$, 500 MHz): δ = 1.56-1.73, 1.93-1.97, 2.06-2.11, 2.29-2.38, 2.40-2.45 (5 m, 3 H, 2 × 1 H, 2 H, 1 H, 3'-H, 4'-H, 5'-H, 6'-H), 2.78 (ddd, *J* = 6.0, 6.2, 12.1 Hz, 1 H, 1'-H), 2.88 (dd, *J* = 10.6, 13.1 Hz, 1 H, 3-H), 2.97 (ddd, *J* = 4.4, 6.2, 10.6 Hz, 1 H, 2-H), 3.10 (dd, *J* = 4.4, 13.1 Hz, 1 H, 3-H), 3.45 (s, 3 H, CO_2Me), 7.01 (d, *J* = 16.1 Hz, 1 H, =CH), 7.13-7.28, 7.36-7.39 (2 m, 4 H, 2 H, Ar), 7.51 (d, *J* = 16.1 Hz, 1 H, =CH), 7.58-7.63 (m, 3 H, Ar) ppm. ^{13}C NMR ($CDCl_3$, 125 MHz): δ = 25.3 (t, C-4'), 27.6 (t, C-5'), 30.8 (t, C-6'), 32.7 (t, C-3), 42.2 (t, C-3'), 46.4 (d, C-1'), 52.6 (d, C-2), 125.8, 126.1, 126.8, 127.1, 127.5, 127.7, 128.8, 130.7, 130.7 (9 d, ArCH=CHPh), 136.6, 137.1, 137.7 (3 s, Ar), 51.5, 175.4 (q, s, CO_2Me), 210.7 (s, C-2') ppm. IR (KBr): $\tilde{\nu}$ = 3060-2860 (=C-H, C-H), 1730, 1710 (C=O), 1600 (C=C) cm^{-1}. MS (EI = 70 eV): *m/z* (%) = 362 (38) $[M]^+$, 264 (39), 205 (100), 178 (23), 159 (15), 115 (49), 91 (83). $C_{24}H_{26}O_3$ (362.5): calcd C 79.53, H 7.23; found C 79.19, H 7.03.

4,4,5,5-Tetramethyl-2-(2-methylpropen-1-yl)-1,3,2-dioxaborolane (SP12): 2-Methylpropen-1-yl nonaflate (0.91 g, 2.57 mmol), bis(triphenylphosphine)palladium(II) dichloride (54 mg, 0.077 mmol), triphenylphosphine (40 mg, 0.15 mmol), potassium phenolate (0.51 g, 3.85 mmol), bis(pinacolato)diborone (0.72 g, 2.83 mmol) and toluene

(4 mL) were treated as described in **GP4** (reaction time 72 h, reaction temperature 70 °C). The obtained yellowish oil was filtered through a plug of silica gel (hexane) and distilled under reduced pressure (bp 69-71 °C/ 25 mbar) to give **SP12** (380 mg, 81%) as a colourless oil. ^{1}H NMR ($CDCl_3$, 270 MHz): δ = 1.26 (s, 12 H, 2 × $OCMe_2$), 1.86, 1.98 (2 s, 2 × 3 H, =CMe_2), 5.12 (s, 1 H, =CH) ppm.

Methyl 2-[2-(2-methylpropen-1-yl)benzyl]-4-oxopentanoate (41): Methyl 2-(2-iodo-benzyl)-4-oxopentanoate (309 mg, 0.89 mmol), bis(triphenylphosphine)palladium(II) dichloride (19 mg, 0.027 mmol), 1,1′-bis(diphenylphosphino)ferrocene (15 mg, 0.027 mmol), potassium carbonate (0.37 g, 2.67 mmol), **SP12** (165 mg, 0.91 mmol) and DMF (4 mL) were treated as described in **GP4** (reaction time 24 h, reaction temperature 75 °C). The obtained yellowish oil was purified by column chromatography (silica gel, hexane/ethyl acetate = 90:10) to give **41** (223 mg, 91%) as a colourless oil. ^{1}H NMR ($CDCl_3$, 500 MHz): δ = 1.68, 1.92 (2 s, 2 × 3 H, =CMe_2), 2.07 (s, 3 H, 5-H), 2.38 (dd, *J* = 4.1, 18.0 Hz, 1 H, 3-H), 2.68 (dd, *J* = 9.4, 13.5 Hz, 1 H, ArCH_2), 2.80 (dd, *J* = 9.6, 18.0 Hz, 1 H, 3-H), 3.00 (dd, *J* = 5.9, 13.5 Hz, 1 H, ArCH_2), 3.10 (dddd, *J* = 4.1, 5.9, 9.4, 9.6 Hz, 1 H, 2-H), 3.63 (s, 3 H, CO_2Me), 6.28 (s, 1 H, =C*H*Ar), 7.08-7.22 (m, 4 H, Ar) ppm. ^{13}C NMR ($CDCl_3$, 125 MHz): δ = 19.3, 26.1 (2 q, =CMe_2), 30.0 (q, C-5), 35.2 (t, Ar*C*H_2), 41.0 (t, C-3), 44.1 (d, C-2), 123.6, 126.4, 126.6, 129.6, 130.4, 136.3, 136.8, 138.2 (5 d, 3 s, ArCH=C), 51.9, 175.5 (q, s, CO_2Me), 206.7 (s, C-4) ppm. IR (neat): $\tilde{\nu}$ = 3090-2855 (=C-H, C-H), 1730, 1715 (C=O), 1655 (br, C=C) cm^{-1}. $C_{17}H_{22}O_3$ (274.4): calcd C 74.42, H 8.08; found C 73.99, H 7.92.
